# Elevated expression of Aurora-A/*AURKA* in breast cancer associates with younger age and aggressive features

**DOI:** 10.1186/s13058-024-01882-x

**Published:** 2024-08-28

**Authors:** L. M. Ingebriktsen, R. O. C. Humlevik, A. A. Svanøe, A. K. M. Sæle, I. Winge, K. Toska, M. B. Kalvenes, B. Davidsen, A. Heie, G. Knutsvik, C. Askeland, I. M. Stefansson, E. A. Hoivik, L. A. Akslen, E. Wik

**Affiliations:** 1 Department of Clinical Medicine, Section for Pathology, Centre for Cancer Biomarkers CCBIO, University of Bergen, Bergen, Norway; 2 Department of Pathology, Haukeland University Hospital, Bergen, Norway; 3 Section for Cancer Genomics, Haukeland University Hospital, Bergen, Norway; 4 Department of Surgery, Section for Breast and Endocrine Surgery, Haukeland University Hospital, Bergen, Norway

## Abstract

**Background and objective:**

Aurora kinase A (*AURKA*) is reported to be overexpressed in breast cancer. In addition to its role in regulating cell cycle and mitosis, studies have reported *AURKA* involvements in oncogenic signaling in suppressing *BRCA1* and *BRCA2*. We aimed to characterize *AURKA* protein and mRNA expression in a breast cancer cohort of the young, investigating its relation to clinico-pathologic features and survival, and exploring age-related *AURKA*-associated biological processes.

**Methods:**

Aurora kinase A immunohistochemical staining was performed on tissue microarrays of primary tumors from an in-house breast cancer cohort (n = 355) with information on clinico-pathologic data, molecular markers, and long and complete follow-up. A subset of the in-house cohort (n = 127) was studied by the NanoString Breast Cancer 360 expression panel for exploration of mRNA expression. METABRIC cohorts < 50 years at breast cancer diagnosis (n = 368) were investigated for differentially expressed genes and enriched gene sets in *AURKA* mRNA high tumors stratified by age. Differentially expressed genes and gene sets were investigated using network analyses and g:Profiler.

**Results:**

High Aurora kinase A protein expression associated with aggressive clinico-pathologic features, a basal-like subtype, and high risk of recurrence score. These patterns were confirmed using mRNA data. High *AURKA* gene expression demonstrated independent prognostic value when adjusted for traditional clinico-pathologic features and molecular subtypes. Notably, high *AURKA* expression significantly associated with reduced disease-specific survival within patients below 50 years, also within the luminal A subtype. Tumors of high *AURKA* expression showed gene expression patterns reflecting increased DNA damage activation and higher BRCAness score.

**Conclusions:**

Our findings indicate higher *AURKA* expression in young breast cancer, and associations between high Aurora-A/*AURKA* and aggressive tumor features, including higher tumor cell proliferation, and shorter survival, in the young. Our findings point to *AURKA* as a marker for increased DNA damage and DNA repair deficiency and suggest *AURKA* as a biomarker of clinical relevance in young breast cancer.

**Supplementary Information:**

The online version contains supplementary material available at 10.1186/s13058-024-01882-x.

## Introduction

The Aurora kinase family consists of the three highly conserved serine-threonine kinases A, B and C (gene names *AURKA*, *AURKB* and *AURKC*), being intracellular enzymes with essential roles during cell division, regulating cell proliferation and growth [1, 2]. Both *AURKA* and *AURKB* are frequently overexpressed in cancer [3, 4], and involved in tumor formation and progression [3, 5].

Aurora kinase A (Aurora-A) is considered as a key oncoprotein in breast cancer progression [6], where the overexpression has been associated with tumor growth, the basal-like phenotype, and poor prognosis [1, 6–8]. The overexpression of Aurora kinase A may be due to gene amplification, as reported in previous studies [1, 7, 9]. *AURKA* was originally named STK15/BTAK (Breast Tumor Amplified Kinase) due to amplification of chromosome 20q13 in breast cancer cell lines, the region where *AURKA* is located [10].

Studies have proposed that overexpression of *AURKA* leads to tumorigenic transformation and DNA instability [11–13], affecting response to cancer therapies [14–16]. *AURKA* is therefore suggested as a promising treatment target [2, 6, 17]. Moreover, in breast cancer, *AURKA* has been reported to outperform the proliferation marker Ki67 as a prognostic marker [18–20]. Until now, *AURKA* has not been investigated in an age-related context.

Here, we investigate the expression of Aurora-A protein/*AURKA* mRNA expression in relation to clinico-pathologic information and outcome, evaluating the prognostic impact in young breast cancer patients, and the relation to biological processes. We discovered that higher Aurora-A positive tumor cell counts (by IHC) and *AURKA* mRNA expression associated with young age at diagnosis, aggressive tumor characteristics, the basal-like phenotype, and high risk of recurrence. Moreover, we found high Aurora-A protein and *AURKA* mRNA expression to be associated with reduced survival, also with independent prognostic impact when adjusting for clinico-pathologic markers and molecular subtypes. Thus, we provide new insights regarding the prognostic significance of Aurora-A/*AURKA* within young breast cancer patients.

## Materials and methods

### Patient cohorts

The study included the Bergen in-house cohort of 355 breast cancer patients aged below 50 years at time of diagnosis, residing in Hordaland County, Norway, and diagnosed with primary invasive breast cancer during the period January 1996-December 2003 [21]. Although we had no information on ethnicity, this population was considered to be homogenous, with few cases of non-European origin in general at the time of inclusion. Prior to immunohistochemistry (IHC), 15 patients were excluded due to missing tissue blocks, and one patient was excluded as only fine needle cytology aspiration was available. This led to 339 cases available for IHC staining. A subset of the in-house cohort (n = 127) was analyzed by the NanoString Breast Cancer 360 panel to obtain mRNA gene expression profiles – see details below. Information on the clinical variables and breast cancer disease were obtained through local pathology registry (Dept. of Pathology, Haukeland University Hospital, Bergen, Norway) and the Cancer Registry of Norway [21]. The follow-up information, acquired from the Norwegian Cause of Death Registry, considered accurate and complete, included information on follow-up time, status at last follow-up, and cause of death. The last date of follow-up was June 30, 2017. Median follow-up time of survivors was 175 months (range 13–257 months).

### Immunohistochemistry analysis

Aurora-A staining was performed manually on 5 μm thin sections from formalin-fixed paraffin-embedded (FFPE) tissue microarray (TMA) blocks with three tumor cores per case [21]. The sections were deparaffinized in xylene, rehydrated through a series of graded alcohols and rinsed in distilled water. Microwave oven heating with Tris–EDTA (Dako/Agilent S1699), pH 6.0, 20 min using 6th sense technology for epitope retrieval. To reduce background staining, a peroxidase-blocking agent (Dako/Agilent S2023) was applied for 8 min before the primary antibody. The tissue sections were incubated at room temperature for 60 min using a monoclonal rabbit antibody against Aurora-A (#91,590) from Cell Signaling Technology, diluted 1:500 before secondary antibody. EnVision-HRP Rabbit (Dako/Agilent K4003) was added for 30 min. To add color at the site of the target antigen recognized by the primary antibody, 3DAB chromogen (Dako/Agilent K3468) was applied for 10 min. Finally, sections were rinsed in distilled water and counterstained with Hematoxylin (Dako/Agilent S3301).

### Aurora-A scoring

All slides were examined and scored by a pathologist (EW), blinded to patient characteristics and outcome. The slides were evaluated using light microscopy (Nikon Eclipse E400) with an eye-piece graticule for counting at × 400 magnification. Care was taken to avoid areas of intense inflammation, fibrosis, necrosis, low cellularity, and poor fixation. The slides were scanned at low magnification (× 100) to identify and encircle the hot-spot defined as the area containing the highest density of Aurora-A positive tumor cells by visual impression. Of the three TMA cores from each tumor, 500 tumor cells were counted in one core. The number of tumor cells and Aurora-A positive tumor cells were recorded, calculating an Aurora-A positivity fraction. For tumors with small areas of invasive tumor (< 500 tumor cells), the total number of tumor cells present were recorded, counting also a 2nd and 3rd core, aiming for a higher tumor cell count (closer to 500). Any nuclear and/or cytoplasmic staining regardless of intensity was considered positive.

### Gene expression resources

For the exploration of gene expression patterns related to *AURKA* in breast cancer, publicly available mRNA gene expression datasets from Molecular Taxonomy of Breast Cancer International Consortium (METABRIC), including information on clinico-pathologic and follow-up data and molecular subtypes, were analyzed (METABRIC discovery cohort, n = 939; METABRIC validation cohort, n = 845) [22]. Information on molecular subtyping based on PAM50 classification was available from original METABRIC study [22]. Expression data was log2-transformed and in cases of multiple probes per gene symbol in the gene expressions matrices, probes were collapsed according to the max probe expression per gene [23]. For valid comparison to our in-house cohort, we applied the same age cutoff (below 50 years) and excluded the normal-like subtype, resulting in two METABRIC age-adjusted cohorts of 204 and 164 patient samples (discovery and validation cohort). Among the 204 cases in the discovery cohort, 53 (26%) were aged below 40 years at diagnosis, while 151 (74%) were at age 40–49 years. In the validation cohort, 54 (33%) were aged below 40 years at diagnosis, while 110 (67%) were at age 40–49 years. In the in-house mRNA cohort (n = 127), 34 (27%) were aged below 40 years at diagnosis, while 93 (73%) were aged 40–49 among the 127 patients.

The online database, “Kaplan–Meier plotter” *(*www.kmplot.com) [24], was used to evaluate *AURKA* expression in relation to recurrence-free breast cancer survival in a merged dataset of Gene Expression Omnibus (GEO) cohorts, (n = 1660). Information of PAM50 molecular subtypes were available within the online tool [25]. Note that METABRIC data is part of the KM-plotter dataset (overlap n = 537/1660) and should therefore be regarded as a semi-independent cohort. For all datasets in this study, the normal-like subtype was excluded from analyses.

### Gene expression data analyses

Differentially expressed genes (DEGs) between *AURKA* high and low tumors (cutoff: median) were identified based on Significance Analysis of Microarrays (SAM) [26]. Gene sets from the Molecular Signatures Database (MsigDB; www.broadinstitute.org/gsea/msigdb) significantly enriched in *AURKA* high tumors were explored by employing Gene Set Enrichment Analysis (GSEA; www.broadinstitute.org/gsea) [23]. The Compute overlaps tool was used to explore gene sets enriched in the identified list of DEGs in *AURKA* high patients. The software J-Express (www.molmine.com) was applied for SAM and GSEA analyses, including assessment of the gene set collections: Gene Ontology (GO)—the category biological function (C5/BP); Hallmark gene sets; Curated gene sets for the KEGG, Reactome, and WikiPathways categories of canonical pathways (C2/CP/KEGG/REACTOME/WIKIPATHWAYS). Based on enriched GO categories, functional characterization of the identified genes was done by use of the Cytoscape app BiNGO [27], showing overrepresented GO categories, adjusted for multiple testing by the Benjamini–Hochberg False Discovery Rate (FDR) correction method. P-values yielded by BiNGO indicates significance illustrated as a gradient from white to orange nodes (darker color represents higher statistical significance).

Functional enrichment analysis based on Gene Ontology (GO) and Kyoto Encyclopedia of Genes and Genomes (KEGG), and Reactome [28–30] was performed using g:Profiler (http://biit.cs.ut.ee/gprofiler/gost) [31].

### In-house gene expression profiling by NanoString

The mRNA gene expression of the 776 genes in the NanoString human BC360 panel was measured in total RNA isolated from formalin-fixed paraffin-embedded (FFPE) breast cancer tissues, using the NanoString nCounter® platform (NanoString Technologies Inc., Seattle, WA, USA). After considering purity and quality requirements (optical density A260/280: 1.75–2.2, A260/230: 1.45–2.2, DV200 > 50%), we obtained RNA profiling for 127 cases (detailed in [32]). For each sample, 300 ng of RNA was hybridized to the BC 360 gene expression panel [33] and signal reads processed by the NanoString nCounter® platform according to manufacturer’s protocol. Pre-processing, quality control, and normalization was performed using nSolver (version 4.0, supported by dependent software R; version 3.3.2; https://cran.r-project.org).

Data were analyzed by the software ROSALIND® (Version 3.35.13.0; https://rosalind.onramp.bio/; San Diego, CA, United States) and nSolver. Fold changes and significance scores were calculated as described in the nCounter® Advanced Analysis 2.0 NanoString User Manual [34]. Significant P-values (P < 0.05) were adjusted for multiple genetic comparisons using the Benjamini–Hochberg method of estimating false discovery rates [35]. Differentially expressed genes (DEGs) between BC in patients aged < 40 and 40–49 years at the time of diagnosis were identified. Volcano plots of differential expression data were plotted using the − log10 (P-value) and log2 fold change.

### PAM50 molecular subtypes and Risk of Recurrence (ROR) score

The 127-sample in-house gene expression dataset was analyzed using the Research Use Only (RUO) version of the NanoString PAM50 algorithm to classify each subject into an intrinsic molecular subtype: luminal A, luminal B, basal-like, and HER2-enriched BCs. This RUO PAM50-based subtype classifier model for the NanoString nCounter Dx Analysis System is consistent with the published qRT-PCR-based PAM50 assay (curated list of 50 genes to distinguish breast tumors into the molecular breast cancer subtypes) [36, 37].

The Prosigna Risk of Recurrence (ROR) score (range 0–100) was calculated by using weighted coefficients to the four molecular subtypes (luminal A, luminal B, HER2-enriched, basal-like), the tumor size (measures dichotomized into ≤ 2.0 cm vs.  > 2.0 cm), and a proliferation score [36–38].

### Statistical methods

Data were analyzed using SPSS (version 25.0, IBM corp., Armonk, NY, USA). Spearman’s rank correlation test was applied when comparing bivariate continuous variables, and Spearman’s correlation coefficients (ρ) were reported. When analyzing differences in distribution of continuous variables between two or more categories, Person’s chi-square, Mann–Whitney U or Kruskal–Wallis tests were applied. For univariate survival analyses, applying recurrence or death from breast cancer endpoints, the Kaplan–Meier product-limit method (log-rank test) was applied. Multivariate breast cancer-specific survival analysis was performed by Cox’ proportional hazards regression model. Variables were included in the Cox survival analyses after evaluating their log-minus-log plot. For multivariate analyses, only patients with information on all variables were included. The calculations were done according to the backward stepwise likelihood ratio test. All statistical tests were two-sided, and statistical significance was assessed at 5% level.

## Results

### High Aurora-A expression associates with aggressive tumor characteristics and young age

**Fig. 1:**
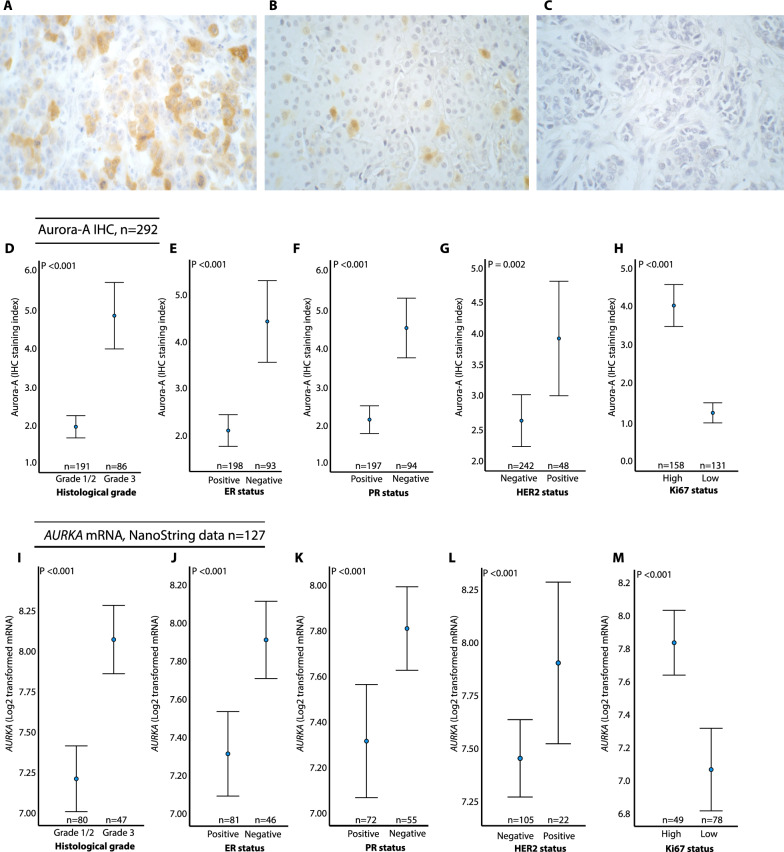
Immunohistochemical staining of Aurora-A, and *AURKA* mRNA/IHC expression across clinico-pathologic characteristics. **A**–**C**: High (**A**), medium (**B**), and low (**C**) proportion of Aurora kinase A (Aurora-A) positive tumor cells. All images magnification × 400. **D**–**H** Aurora-A expression across histological grade (**D**), ER status (**E**), PR status (**F**), HER2 status (**G**), and Ki67 status (**H**). **I**–**M** *AURKA* mRNA expression across histological grade (**I**), ER status (**J**), PR status (**K**), HER2 status (**L**), and Ki67 staus (**M**). Data shown with error-bars representing 95% confidence interval of the mean, and *P*-values by Mann–Whitney U-test. Expression values are displayed as IHC staining index and Log2 transformed mRNA expression values. All data from the in-house cohort

When performing Aurora-A immunohistochemistry staining on our in-house FFPE tissue microarray cohort from primary breast cancer, Aurora-A showed cytoplasmic and/or nuclear staining with varying intensity and proportion of positive tumor cells (Fig. 1A–C**)**. The proportion of Aurora-A positive tumor cells ranged from 0 to 93% (median 10%). In the in-house cohort, we demonstrated a significant association between *AURKA* gene expression and Aurora-A protein (IHC) positive tumor cell fraction (*P* = 0.004, *ρ* = 0.70, data not shown).

When investigating how the Aurora-A protein expression was related to clinico-pathologic variables, we found associations between high Aurora-A IHC positive tumor cell counts and high histologic grade, ER and PR negativity, HER2 positivity, and high Ki67 (in-house cohort; Fig. 1D–H**, **Table 1). No significant associations were observed between the levels of Aurora-A positivity and tumor size, nor lymph node status. In the in-house NanoString mRNA expression cohort and external METABRIC discovery and validation cohorts (< 50 cohorts, n = 368), we observed associations between high *AURKA* mRNA expression and high histologic grade, lymph node metastases, ER negativity, and high Ki67 (Fig. 1I–M; Supplementary Fig. 1A-H). In the METABRIC validation cohort, we also found an association between high *AURKA* mRNA and large tumor size (Supplementary Fig. 1I).

**Table 1:** Aurora-A positive tumor cell fraction (%, by IHC) across tumor characteristics and breast cancer subtypes

Aurora-A	n (n%)	n (n%)	n (n%)	n (n%)	*P*-value
	Q1	Q2	Q3	Q4	
*Aurora-A IHC in-house cohort (n = 292)*					
*Age*					
< 40	17 (23.6)	12 (16.7)	20 (27.8)	23 (31.9)	NS
40–49	63 (28.8)	53 (24.2)	53 (24.2)	50 (22.8)	
*Tumor size*					
≤ 20 mm	48 (29.5)	35 (21.5)	44 (26.9)	36 (22.1)	NS
> 20 mm	31 (25.0)	28 (22.6)	28 (22.6)	37 (29.8)	
*LN status*					
Negative	48 (31.8)	30 (19.9)	35 (23.2)	38 (25.1)	NS
Positive	31 (22.8)	33 (24.3)	37 (27.2)	35 (25.7)	
*Histologic grade*					
1 or 2	66 (34.6)	51 (26.7)	48 (25.1)	26 (13.6)	** < 0.001**
o3	9 (10.5)	12 (14.0)	21 (24.4)	44 (51.1)	
*ER status*					
Positive	65 (32.8)	49 (24.8)	50 (25.3)	34 (17.1)	** < 0.001**
Negative	15 (16.1)	16 (17.2)	23 (24.7)	39 (42.0)	
*PR status*					
Positive	64 (32.5)	55 (27.9)	48 (24.4)	30 (15.2)	** < 0.001**
Negative	16 (17.0)	10 (10.6)	25 (26.6)	43 (45.8)	
*HER2 status*					
Positive	8 (16.7)	7 (14.6)	15 (31.2)	18 (37.3)	**0.035**
Negative	72 (29.7)	58 (24.0)	58 (24.0)	54 (22.3)	
*Molecular subtypes IHC*					
Luminal A	53 (46.9)	34 (30.1)	20 (17.7)	6 (5.3)	** < 0.001**
Luminal B	14 (16.5)	18 (21.2)	29 (34.1)	24 (28.2)	
Luminal B HER2 +	4 (16.0)	7 (28.0)	6 (24.0)	8 (32.0)	
HER2 + non Luminal	4 (17.4)	0 (0)	9 (39.1)	10 (43.5)	
Triple Negative	5 (11.7)	6 (13.7)	9 (20.5)	24 (54.1)	
Molecular subtypes by PAM50*					
Luminal A	14 (34.1)	15 (36.6)	5 (12.2)	7 (17.1)	**0.003**
Luminal B	8 (28.6)	4 (14.2)	8 (28.6)	8 (28.6)	
HER2-Enriched	2 (9.0)	3 (13.5)	8 (36.6)	9 (40.9)	
Basal-like	2 (7.7)	3 (11.5)	8 (30.8)	13 (50.0)	
Risk groups*					
Low risk	8 (47.0)	6 (35.3)	1 (5.9)	2 (11.8)	**0.004**
Intermediate risk	7 (26.9)	8 (30.8)	5 (19.2)	6 (23.1)	
High risk	11 (14.9)	11 (14.9)	23 (31.0)	29 (39.2)	
*Ki67 status*					
Low	60 (45.8)	37 (28.2)	26 (19.9)	8 (6.1)	** < 0.001**
High	19 (12.0)	27 (17.1)	47 (29.7)	65 (41.2)	

*PAM50 subtypes and risk groups for n = 117 samples

Missing: age n = 1, tumor size n = 5, lymph node status (LN) n = 5, Ki67 status = 3

Histologic grade n = 15, ER status n = 1, PR status n = 1,

HER2 status n = 2, molecular subtypes by immunohistochemistry (IHC) n = 2

Q1-4 = Aurora-A quartiles 1–4

**Fig. 2:**
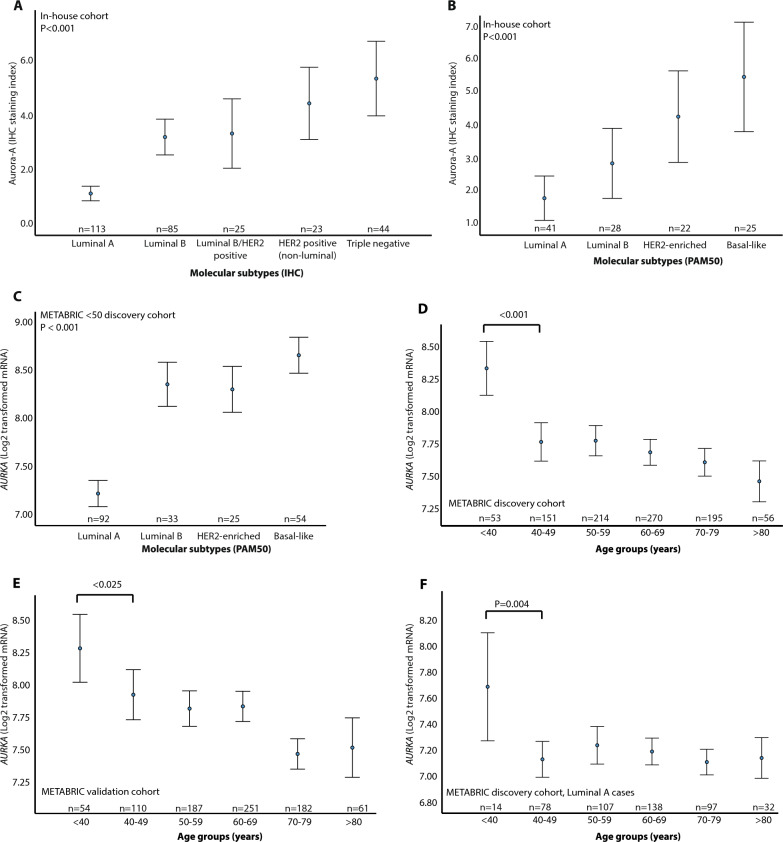
Aurora kinase A (*AURKA*) expression associates with aggressive subtypes and young age. **A** Aurora kinase A (Aurora-A) expression across molecular subtypes (by IHC; in house cohort, n = 292), **B** molecular subtypes by PAM50 (in-house cohort, n = 116). **C** *AURKA* mRNA expression across molecular subtypes (by PAM50; METABRIC < 50 cohort, n = 204), **D** and age groups in METABRIC discovery cohort (n = 939), **E** METABRIC validation cohort (n = 845), **F** and METABRIC discovery cohort with luminal A cases only (n = 466). Data shown with error-bars representing 95% confidence interval of the mean, and *P*-values by Mann-Witney U-test

When examining how Aurora-A protein and *AURKA* mRNA expression was distributed across molecular subtypes, we observed the highest levels of Aurora-A IHC positive tumor cells and *AURKA* mRNA expression in the triple negative and basal-like subtype in both the in-house and METABRIC cohorts (< 50 cohorts, n = 368), and high *AURKA* levels also in the luminal B and HER2 positive/enriched subtypes (Fig. 2A–C**; **Table 1). Moreover, when examining the distribution of Aurora-A IHC and mRNA levels across risk of recurrence groups, we found associations between high Aurora-A/*AURKA* levels and a high-risk score (Supplementary Fig. 2A-B).

When investigating how *AURKA* expression varied between age groups, we observed a successive decline in *AURKA* expression towards age-groups of increasing 10-year intervals. The largest stepwise difference in *AURKA* mRNA expression was observed comparing patients under 40 years to the patient group aged 40–49 years (Fig. 2D–E**;** METABRIC combined cohorts, all ages, n = 1784). Also, within the luminal A cases, we found significantly higher *AURKA* mRNA expression among patients below 40 years compared to those aged 40–49 (Fig. 2F; METABRIC discovery cohort). In the in-house cohort, we observed higher expression of *AURKA* mRNA in the below 40 group, however, the same result was not observed for Aurora-A IHC data (Supplementary Fig. 2C-D).

### Aurora-A expression presents independent prognostic value

**Fig. 3:**
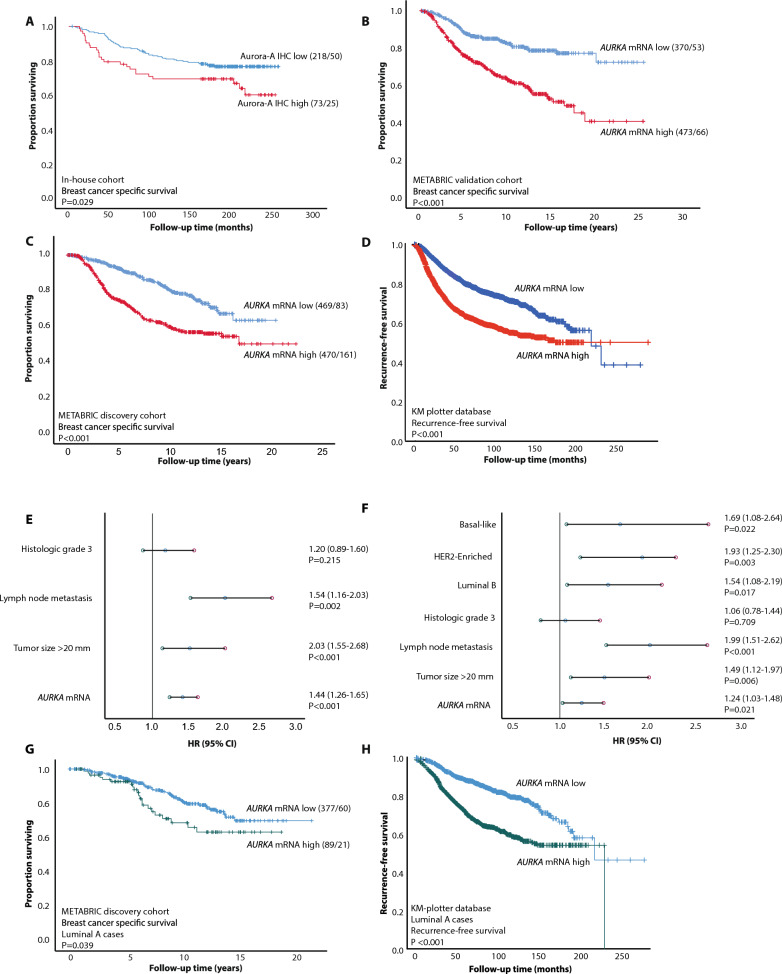
High *AURKA *expression associates with poor survival. **A** High Aurora-A expression associates with shorter disease-specific survival (n = 291; in-house cohort). **B**–**C** High *AURKA* mRNA expression associates with shorter disease-specific survival in METABRIC validation- (**B**; n = 843) and discovery cohort (**C**; n = 939) cohort. (**D**) Recurrence-free breast cancer survival according to *AURKA* mRNA in the cohorts from the online KM plotter database (n = 4929; www.kmplot.com). **E**–**F** When adjusting for traditional prognostic variables, *AURKA* mRNA demonstrated independent association with shorter disease-specific survival (**E**; Cox multivariate analysis) also when adding molecular subtypes to the analysis (**F**). The lines represent a hazard ratio (HR) of 1.0, and error bars represent 95% CI. **G**–**H** High *AURKA* expression was also significantly associated with shorter survival in luminal A tumors (METABRIC discovery and KM plotter cohorts, n = 466 and n = 2277 respectively). KM plots: numbers in brackets indicate number of patients/number of events

We found that high levels of Aurora-A IHC positive tumors cells and high *AURKA* mRNA expression were associated with shorter disease-specific survival (Fig. 3A–C). High *AURKA* expression also predicted recurrence-free survival in the breast cancer KMplotter cohort [24]; Fig. 3D). By adding the clinico-pathologic variables tumor diameter, histologic grade, and lymph node status to the multivariate analysis (METABRIC discovery cohort, all ages, n = 1784), *AURKA* mRNA demonstrated independent association with shorter disease-specific survival (*P* < 0.001**,** HR = 1.44 95% CI 1.26–1.65, Fig. 3E). When additionally including molecular subtypes to the Cox model, *AURKA* mRNA expression maintained independent association with reduced survival (*P* = 0.021, HR = 1.24 95% CI 1.03–1.48, Fig. 3F).

Upon investigating the prognostic impact of *AURKA* expression in individual molecular breast cancer subtypes, we found that high *AURKA* expression was associated with reduced disease-specific and recurrence-free survival in luminal A tumors (Fig. 3G–H; METABRIC discovery (all ages, n = 1784) and KM-plotter data, respectively). Moreover, high *AURKA* mRNA expression also showed independently significant prognostic value in luminal tumors, when adjusting for the traditional clinico-pathologic variables and MKI67 mRNA expression (Table 2A**;** METABRIC discovery cohort). In luminal A tumors only, *AURKA* mRNA presented independent prognostic value in addition to MKI67 mRNA expression, when adjusting for the traditional clinico-pathologic variables (Table 2B).

**Table 2:** Uni- and multivariate analysis. Cox' proportional hazards regression with disease-specific death from breast cancer as end-point. METABRIC discovery cohort luminal A and B subtypes combined (n = 734) and luminal A subtype only (n = 466)

Variables	n	n (%)	Univariate HR (95% CI)	*P*-value	Multivariate HR (95% CI)	*P*-value
*(A) METABRIC discovery luminal A and B subtypes combined (n = 734)*						
Histologic grade						
Grade 1 or 2	435	59	1	** < 0.001**	1	NS
Grade 3	299	41	1.69 (1.24–2.29)		1.10 (0.79–1.53)	
Tumor diameter						
< 20 mm	329	45	1	** < 0.001**	1	** < 0.001**
> 20 mm	405	55	2.21 (1.60–3.06)		1.83 (1.31–2.55)	
Nodal status						
Negative	400	55	1	** < 0.001**	1	** < 0.001**
Positive	334	45	2.17 (1.60–2.96)		1.90 (1.38–2.62)	
MKI67	734		3.19 (2.00–5.10)	** < 0.001**	2.22 (1.25–3.95)	**0.012**
* AURKA* mRNA	734		1.56 (1.32–1.84)	** < 0.001**	1.30 (1.06–1.60)	**0.007**
*(B) METABRIC discovery luminal A subtype only (n = 466)*						
Histologic grade						
Grade 1 or 2	333	71	1	NS	1	NS
Grade 3	133	29	1.52 (0.97–2.39)		1.22 (0.92–1.63)	
Tumor diameter						
< 20 mm	232	49	1	** < 0.001**	1	**0.003**
> 20 mm	234	51	2.28 (1.45–3.59)		1.50 (1.15–2.30)	
Nodal status						
Negative	273	59	1	**0.006**	1	** < 0.001**
Positive	193	41	1.84 (1.19–2.86)		2.09 (1.60–2.73)	
MKI67	466		4.19 (1.47–11.97)	**0.007**	1.59 (1.06–2.37)	**0.027**
* AURKA* mRNA	466		1.48 (1.14–1.93)	**0.004**	1.31 (1.11–1.54)	**0.001**

HR = Hazard ratio, CI = Confidence interval, n = number of patients

**Fig. 4:**
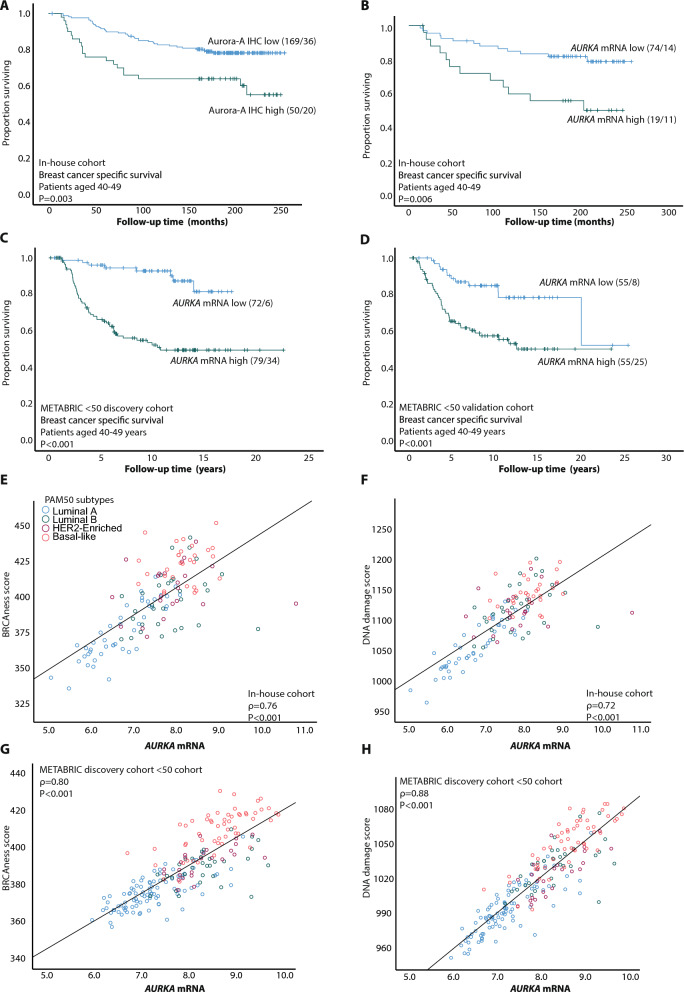
Aurora kinase A IHC and *AURKA* mRNA associates with poor survival in patients aged 40–49, and *AURKA* mRNA correlates with BRCAness- and DNA damage score. **A**–**B** Aurora-A and *AURKA* mRNA expression associated with shorter disease-specific survival in patients aged 40–49 years (n = 219 and n = 93 respectively, in-house cohort). **C**–**D** High *AURKA* mRNA expression was also significantly associated with shorter survival in patients aged 40–49 years in METABRIC < 50 discovery (**C**; n = 151) and validation (**D**; n = 110) cohorts. **E**–**H** Correlation between BRCAness score and DNA damage score and *AURKA* mRNA (**E**–**F**; in-house cohort, **G**–**H**; METABRIC < 50 discovery cohort). Scatter plots are represented with P-values by Spearman's rank correlation and the corresponding coefficients (ρ). Gene expression values are Log2-transformed mRNA levels. KM plots: numbers in brackets indicate number of patients/number of events. BRCAness- and DNA damage score calculated from Log2 transformed mRNA

When investigating the prognostic impact of Aurora-A protein and mRNA expression separately for patients below 40 and 40–49 years, we found that both high Aurora-A protein and *AURKA* mRNA expression associated with reduced survival in patients aged 40–49 (Fig. 4A–D) but did not show prognostic value in the patient group below 40 years of age (data not shown). To note, when adding the clinico-pathologic variables tumor diameter, histologic grade, lymph node status, and MKI67 to the multivariate analysis (< 50 METABRIC discovery and validation cohorts, n = 368), *AURKA* mRNA demonstrated independent association with shorter disease-specific survival (P < 0.001**,** HR = 2.25 95% CI 1.63–3.10 and HR = 2.21 95% CI 1.50–3.26 respectively) for patients aged 40–49 years (Supplementary Table 1).

### Gene expression profiles in young breast cancer with high *AURKA* expression reflect pathways related to proliferation and DNA damage

To study the potential age-related differences of biological processes accompanying alterations in *AURKA* mRNA expression in breast cancer, we analyzed global gene expression data from primary tumors in breast cancer patients. We employed the combined METABRIC discovery and validation cohorts for this purpose (all ages, n = 1784).

When examining genes differentially expressed between *AURKA* expression high and low, we identified 178 upregulated and 173 downregulated genes in the *AURKA*-high group (Supplementary Table 2; fold change ≥ 1.5/ ≤  − 1.5, FDR < 0.008%; METABRIC cohorts). As expected, we observed multiple cell-cycle-related genes among the top-ranked upregulated genes: UBE2C, CDC20, AURKB, and CCNB2 (Supplementary Table 2). In line with this observation, signatures reflecting proliferation pathways were in gene set enrichment analyses repeatedly enriched in *AURKA* mRNA-high tumors (Supplementary Table 3; GSEA, MsigDB; FDR < 5%).

Next, we investigated enriched gene sets within *AURKA*-high tumors, age-stratified, comparing output from GSEA analysis on *AURKA* high vs low between patients aged below 40 years (n = 58) and patients aged 40–49 (n = 151) (METABRIC discovery < 50 cohort, n = 204); MSigDB/Hallmark gene sets; KEGG; GO/Biological processes). Gene sets reflecting proliferation were top enriched in both age groups (Supplementary Table 4). We age-stratified the analyses of genes differentially expressed between *AURKA* high and low tumors, searching for *AURKA*-high associated genes uniquely up- and downregulated in the young and older patients (METABRIC < 50 cohorts, n = 368), fold change ± 1.5, FDR < 5%). Within the patient group aged below 40 years, we identified 75 unique upregulated genes and 72 unique downregulated genes in the *AURKA*-high subset. For patients 40–49 years, 51 genes were uniquely upregulated, and 86 genes uniquely downregulated in *AURKA*-high tumors (Supplementary Fig. 3, Supplementary Table 5).

When exploring functional enrichment from the gene list associated with high *AURKA* expression in the young, using the g:Profiler analysis tool, the top ranked GO/biological processes uniquely enriched in the *AURKA*-high/young group related to DNA replication, cell cycle, and DNA duplex unwinding (Supplementary Table 6). Moreover, gene sets reflecting activity in DNA replication and the cell cycle were top ranked pathways activated within the KEGG and Reactome databases, respectively, and were uniquely enriched in the *AURKA*-high/young group. The unique DEGs downregulated in the *AURKA*-high/young group showed enrichment of functions related to extracellular matrix organization, and extracellular structure organization (Supplementary Table 6).

Based on the uniquely up-regulated DEGs within *AURKA-* high tumors from patients aged below 40 years, we constructed a protein–protein interaction network assessing the overrepresentation of gene ontology categories (GO: Biological processes) among these genes. We demonstrated enrichment of GO categories reflecting cell-cycle, DNA replication, double strand break repair and DNA repair (Supplementary Fig. 4; Supplementary Table 7; *P* < 0.001).

Due to our results demonstrating *AURKA* mRNA being independent in multivariate analysis against Ki67, we wanted to explore whether Ki67 and AURKA mRNA shared common categorical gene set enrichment, and whether there were any unique enrichments associated with *AURKA* mRNA. In analyses on gene sets enriched in Ki67-high tumors, the results were much alike the output for *AURKA*-high tumors—gene sets reflecting proliferation dominated the top-ranked list (Supplementary Table 8). When comparing genes differentially expressed between Ki67-high and -low tumors (Supplementary Table 9) with genes differentially expressed between *AURKA* high and low cases (Supplementary Table 2), we found nine and seven uniquely differentially up- and downregulated genes in Ki67-high tumors (Supplementary Fig. 5). Several of the uniquely upregulated genes are previously shown to be involved in tumor cell proliferation such as *UBE2C* [39], *GABRP* [40], and *FOXC1* [41].

### Patients with high *AURKA* expression have increased DNA damage activation and BRCAness score

To further investigate the enrichment of gene sets reflecting DNA damage and repair in the young *AURKA*-high group, we investigated how *AURKA* mRNA expression related to DNA damage- and BRCAness scores (see MM section), stratified by age. We found strong correlations between *AURKA* mRNA expression and both signature scores (Fig. 4E-H; Supplementary Fig. 2H-I; In-house and METABRIC < 50 cohorts, n = 368), also when examined independently in the age groups < 40 and 40–49 (Supplementary Fig. 6A-L). When investigating the DNA damage score and BRCAness score across these age groups, and stratified for molecular subtypes, we found higher expression of both scores in luminal A subtypes (METABRIC discovery cohort; Supplementary Fig. 6M-N).

## Discussion

Studies based on Aurora-A protein- and *AURKA* mRNA expression in breast cancer have implied that high Aurora kinase A expression is a strong and independent prognostic marker [42–45]. However, data on whether Aurora-A/*AURKA* expression is associated with prognosis specifically in young breast cancer has been lacking. In this study, we aimed to characterize Aurora-A/*AURKA* in young breast cancer patients and evaluate its prognostic significance. To our knowledge, this is the first study describing the expression and prognostic value of Aurora-A protein in patients aged below 50 years, paired with well-characterized clinico-pathologic variables and long and complete follow-up information.

We demonstrated higher expression of Aurora-A in tumors from the youngest breast cancer patients (below 40 years) compared to the older (40-49 years), emphasizing that high levels of Aurora-A kinase protein and *AURKA* mRNA expression point to aggressive tumor features and associate with reduced survival in breast cancer patients below 50 years. The Aurora-A/*AURKA* expression levels demonstrated independent prognostic impact also when adjusting for the traditional clinico-pathologic markers and molecular subtypes, also in the age group 40–49 years, as has been demonstrated in studies by others, but not specifically in young breast cancer cohorts [16, 44]. Of note, a previous study on Ki67 from our group showed lower prognostic impact of Ki67 in the young compared to older [46].

Overexpression of Aurora-A may lead to tumorigenic transformation and DNA instability [11–13]. Notably, increased expression of Aurora-A also promotes cell cycle progression despite abnormal chromosomal segregation, even when DNA is damaged, which is known as a hallmark of malignant tumors [47]. By multiple analytical approaches, our results provide evidence for increased tumor cell proliferation in breast cancer in *AURKA*-high tumors, in line with previous studies from our group and others [21, 48–52].

When comparing differentially expressed genes and enriched gene sets between *AURKA*-high and Ki67-high tumors, we observed very similar outputs, with gene sets reflecting proliferation dominating the top-ranked enriched list. Among the uniquely differentially up- and downregulated genes in Ki67-high tumors, several of these are previously shown to be involved in tumor cell proliferation, suggesting that both Ki67 and *AURKA* are contributes to tumor cell proliferation, potentially with involvement in different pathways and biological processes contributing to tumor growth.

Due to the crucial functions of aurora kinases in the cell cycle, particularly in the G2-M phases, it is expected that their effects will be affected following DNA damage, aiming to maintain the DNA checkpoint functionality [4]. Studies have shown that overexpression of Aurora-A can invalidate the G2 DNA damage checkpoint, and that high Aurora-A expression may lead to initiation of G2-M transition via the CDC25, p53 and PLK1 pathways [53–55]. Also, abnormal expression of Aurora-A may cause aneuploidy, which in turn can lead to an accumulation of defect or abnormal cells which ultimately contributes to malignancy [56]. These studies demonstrate a link between high levels of Aurora-A and increased DNA damage in cancer. Our data supports this, suggesting *AURKA* as a marker of increased DNA damage and concurrently deficient DNA repair, also observed in the luminal A subset in the young (age below 40 years).

In conclusion, our results demonstrate associations between high Aurora-A/*AURKA* expression and young age, as well as with aggressive tumor features including increased tumor cell proliferation. Also, increased DNA damage and DNA repair deficiency in *AURKA*-high tumors is indicated. Our findings point to *AURKA* as a biomarker relevant for young breast cancer patients.

### Supplementary Information

Additional file 1.

Additional file 2.

Additional file 3.

Additional file 4.

Additional file 5.

Additional file 6.

Additional file 7.

Additional file 8.

Additional file 9.

Additional file 10.

Additional file 11.

Additional file 12.

Additional file 13.

Additional file 14.

Additional file 15.

## Data Availability

The METABRIC gene expression datasets are available at https://ega-archive.org/studies/EGAS00000000083 (METABRIC).
