# Supplementary material for: Elevated expression of Aurora-A/AURKA in breast cancer associates with younger age and aggressive features
Source: Breast Cancer Res. 2024 Aug 28;26:126. doi: 10.1186/s13058-024-01882-x (PMC11360479; doi:10.1186/s13058-024-01882-x)
Supplement: Supplementary file 1 — Additional file 1. [file 13058_2024_1882_MOESM1_ESM.pdf]

Supplementary Figure 1

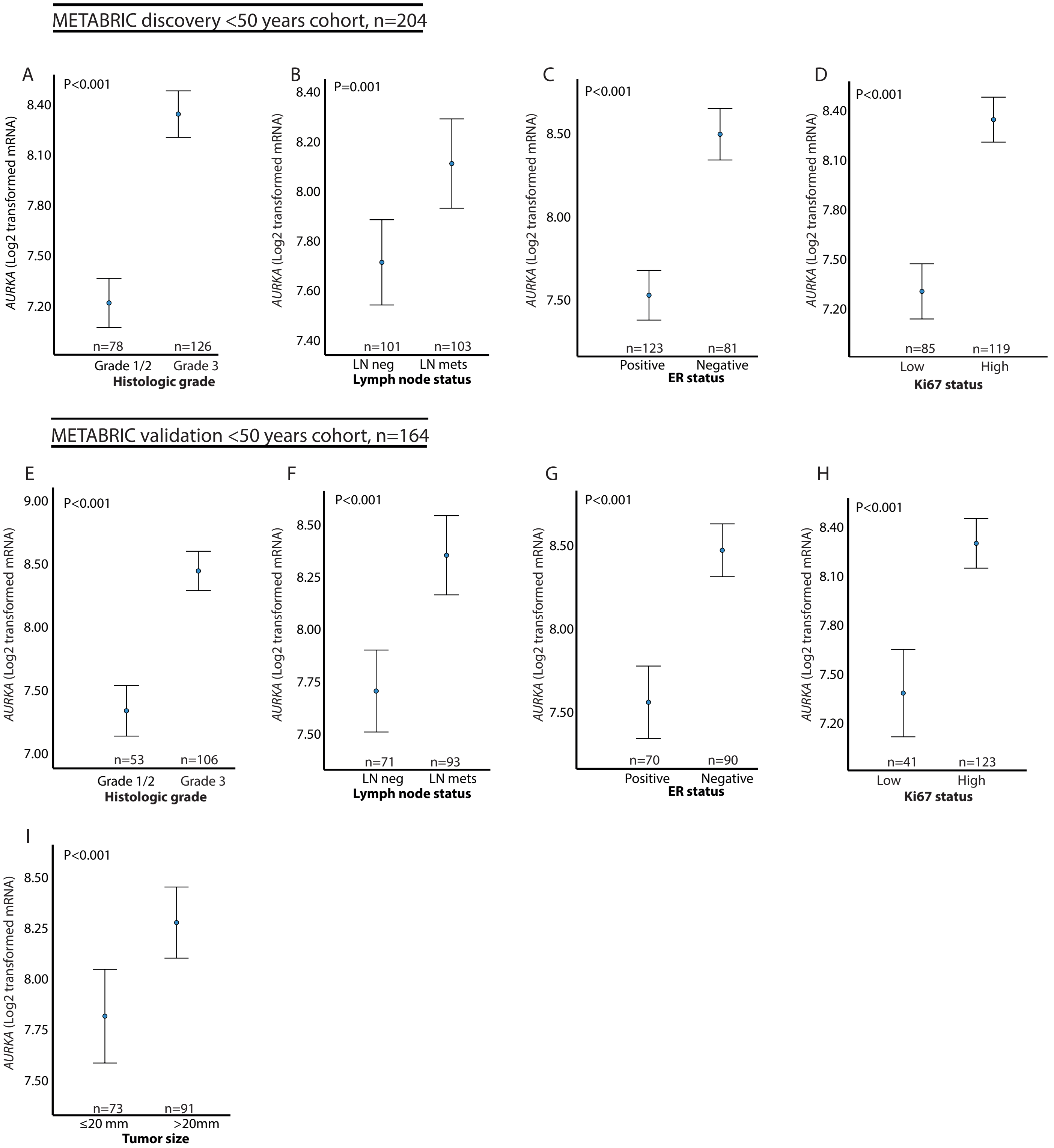

Supplementary Figure 1: ***AURKA* mRNA expression acorss clinico-pathologic characteristics**.
(**A-H**) *AURKA* mRNA across histologic grade, lymph node status, ER status, and Ki67 status (A-D; METABRIC <50 discovery cohort n=204, E-H; METABRIC <50 validation cohort n=164). (**I**) *AURKA* mRNA across tumor size (METABRIC <50 validation cohort n=164). Data shown with error-bars representing 95% confidence interval of the mean, and P-values by Mann-Whitney U-test. Gene expression values are displayed as Log2-transformed mRNA levels.
