# Supplementary material for: Elevated expression of Aurora-A/AURKA in breast cancer associates with younger age and aggressive features
Source: Breast Cancer Res. 2024 Aug 28;26:126. doi: 10.1186/s13058-024-01882-x (PMC11360479; doi:10.1186/s13058-024-01882-x)
Supplement: Supplementary file 2 — Additional file 2. [file 13058_2024_1882_MOESM2_ESM.pdf]

Supplementary Figure 2

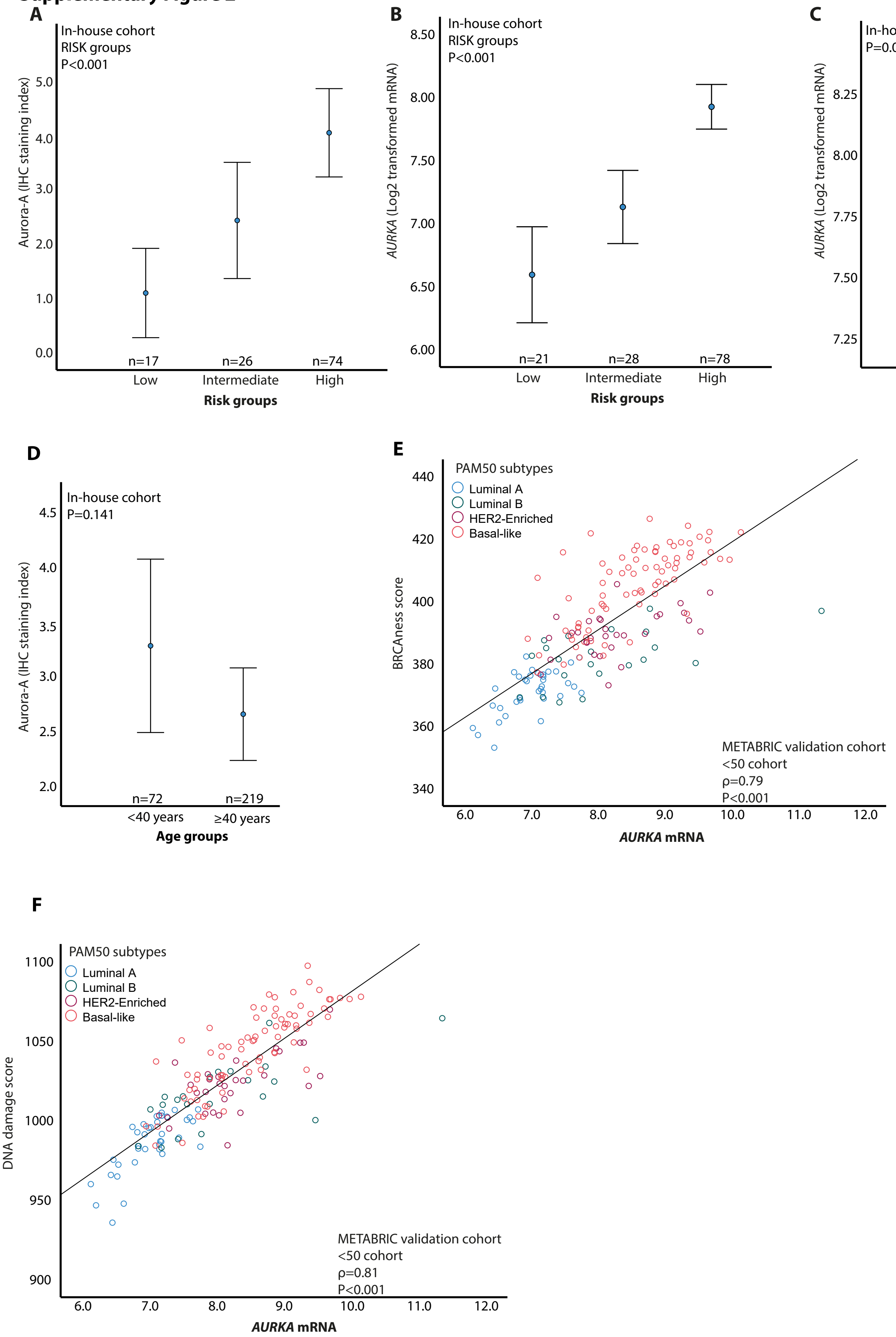

Supplementary Figure 2: Aurora-A and *AURKA* mRNA associates with high risk, young age, and *AURKA* mRNA correlates with BRCAness- and DNA damage score.

(**A-B**) Aurora-A and *AURKA* mRNA across risk groups (in-house cohort, n=117). (**C-D**) *AURKA* mRNA and Aurora-A across age (in-house cohort, n=127 and n=292 respectively). (**E-F**) Correlation between BRCAness- (E) and DNA damage score (F) and *AURKA* mRNA (METABRIC <50 validation cohort, n=164. Data shown with error-bars representing 95% confidence interval of the mean, and p-values by Mann-Whitney U-test. Scatter plots are presented with P-values by Spearman's rank correlation and the corresponding coefficients (ρ). Gene expression values are displayed as Log2-transformed mRNA levels. BRCAness score and DNA damage score are calculated from Log2 transformed mRNA.
