# Supplementary material for: Elevated expression of Aurora-A/AURKA in breast cancer associates with younger age and aggressive features
Source: Breast Cancer Res. 2024 Aug 28;26:126. doi: 10.1186/s13058-024-01882-x (PMC11360479; doi:10.1186/s13058-024-01882-x)
Supplement: Supplementary file 3 — Additional file 3. [file 13058_2024_1882_MOESM3_ESM.pdf]

Supplementary Figure 3

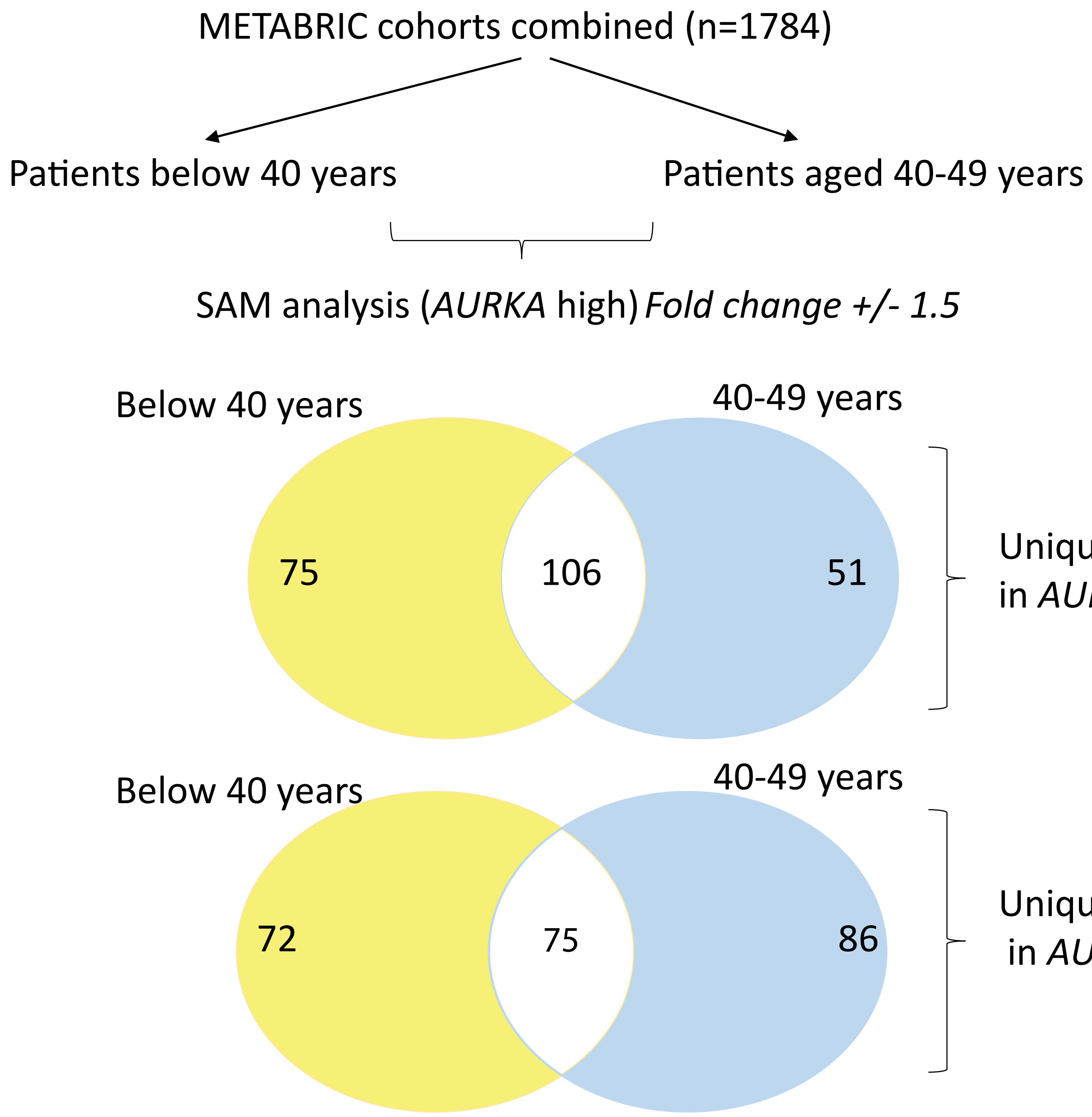

Supplementary Figure 3: The workflow from analyses of the METABRIC cohorts (n = 1784).
SAM analysis revealed 75 and 51 uniquely upregulated differentially expressed genes (DEGs) in patients aged below 40 and 40-49 years respectively in *AURKA* expression high with fold change >1.5/<1.5. 72 and 86 uniquely downregulated DEGs were revealed in patients aged below 40 and 40-49 years respectively in *AURKA* expression high with fold change >1.5/<1.5.
