# Supplementary material for: Elevated expression of Aurora-A/AURKA in breast cancer associates with younger age and aggressive features
Source: Breast Cancer Res. 2024 Aug 28;26:126. doi: 10.1186/s13058-024-01882-x (PMC11360479; doi:10.1186/s13058-024-01882-x)
Supplement: Supplementary file 4 — Additional file 4. [file 13058_2024_1882_MOESM4_ESM.pdf]

Supplementary Figure 4

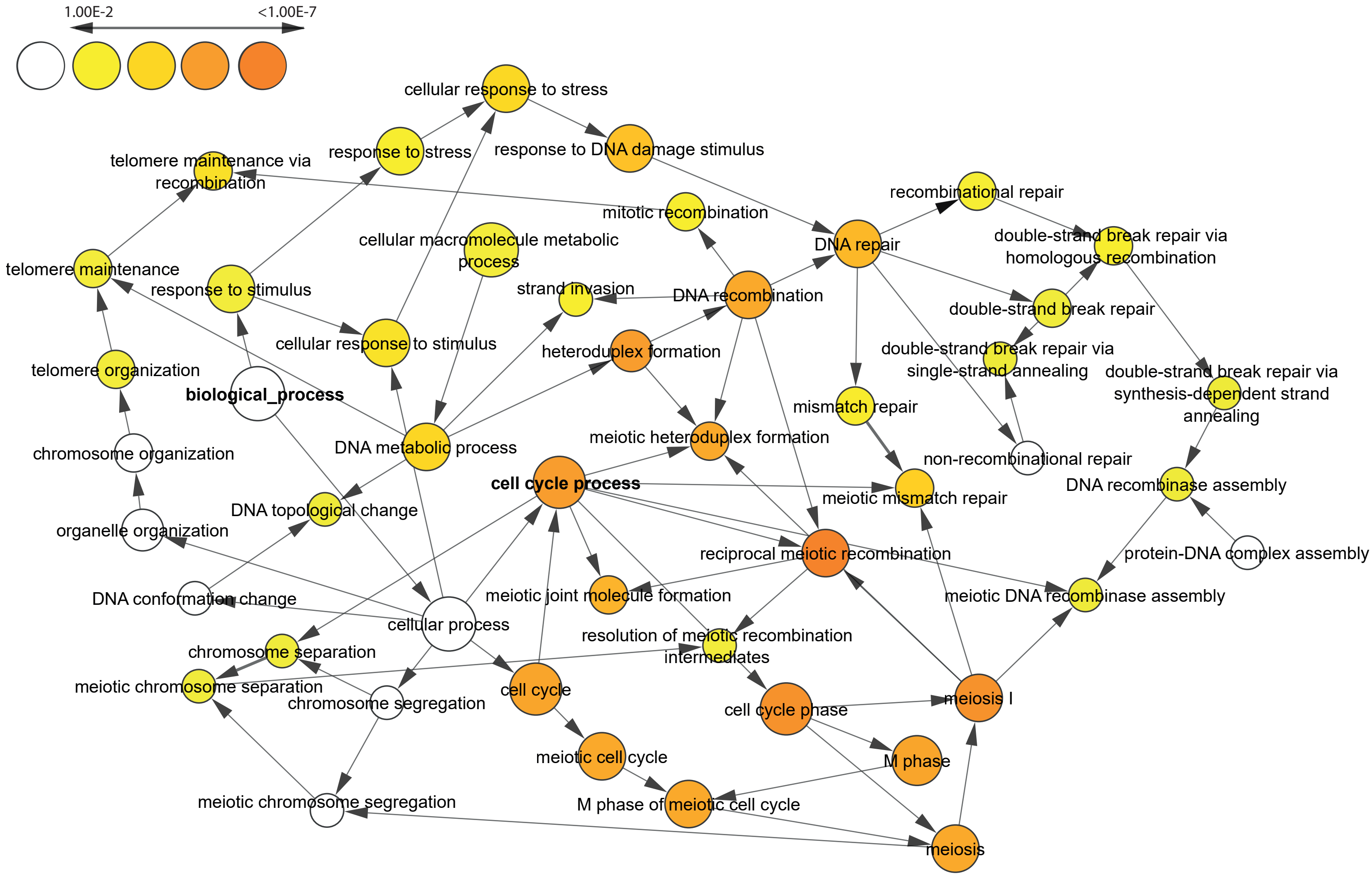

Supplementary Figure 4: Visualization of Gene Ontology (GO) biological processes (BP) performed by the Cytoscape App BiNGO. The size of a node indicates the number of genes enriched in this term. The color represented its P value, the smaller the P value, the darker the node is. The arrows represent progression of BP terms.
