# Supplementary material for: Elevated expression of Aurora-A/AURKA in breast cancer associates with younger age and aggressive features
Source: Breast Cancer Res. 2024 Aug 28;26:126. doi: 10.1186/s13058-024-01882-x (PMC11360479; doi:10.1186/s13058-024-01882-x)
Supplement: Supplementary file 5 — Additional file 5. [file 13058_2024_1882_MOESM5_ESM.pdf]

Supplementary Figure 5

Comparison between the 50 top ranked genes differentially expressed (DEGs) between AURKA high vs low (cut-point upper quartile) and Ki67 high vs low (cut-point upper quartile). Nine and seven genes were uniquely up- and downregulated in Ki67-high tumors. Cut point for both SAM analyses Fold change ≥1.5 / ≤ -1.5; FDR <0.008.

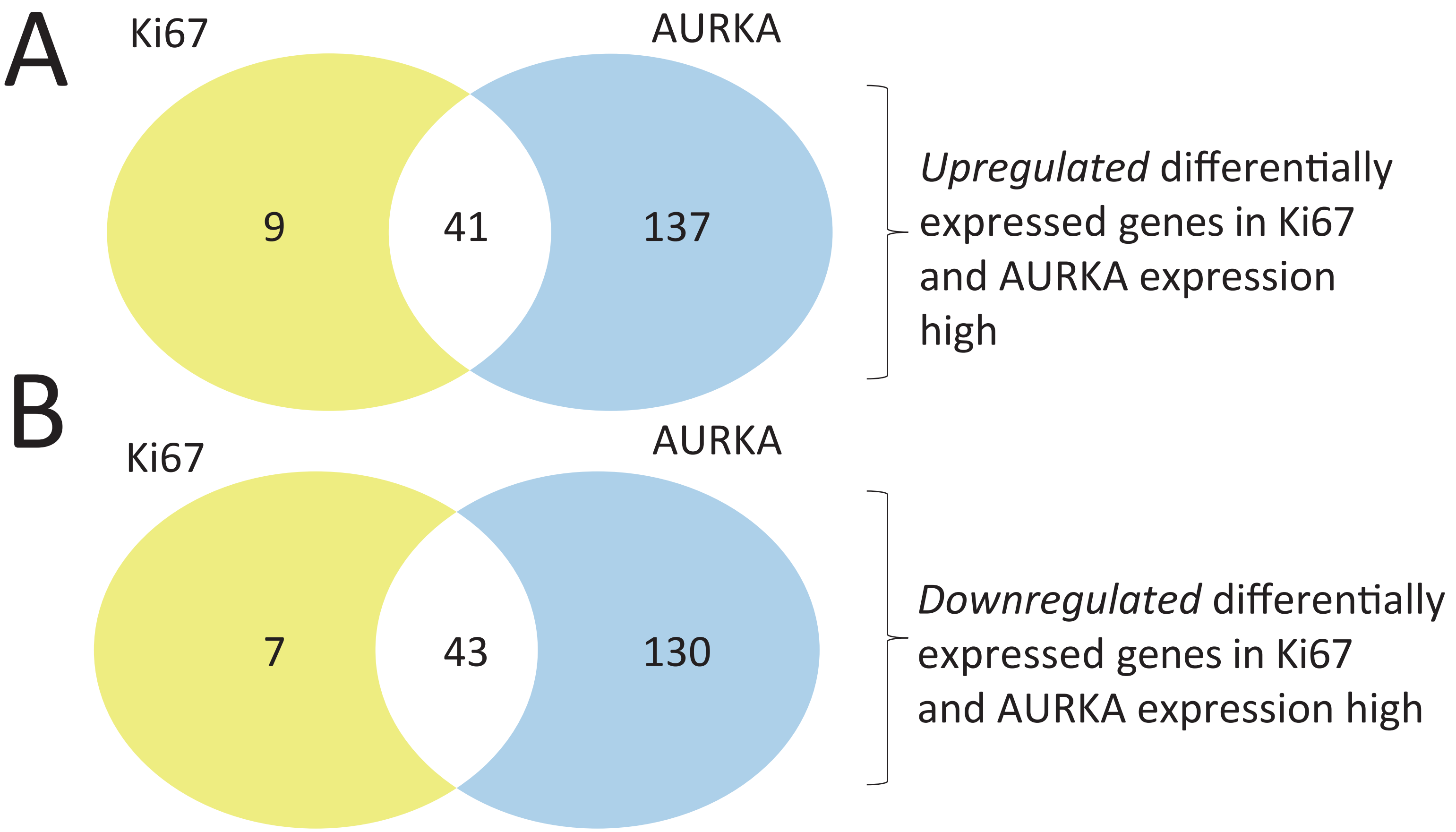

9 Unique upregulated DEGs in Ki67 expression high:
FOXC1 CRABP1 GABRP ELF5 ROPN1 PROM1 UBE2C FAM171A1 BCL11A

7 unique downregulated DEGs in Ki67 expression high*:*
TPSAB1 FSIP1 CAPN13 SERPINA5 AGR2 COL10A1 EEF1A2
