# Supplementary material for: Elevated expression of Aurora-A/AURKA in breast cancer associates with younger age and aggressive features
Source: Breast Cancer Res. 2024 Aug 28;26:126. doi: 10.1186/s13058-024-01882-x (PMC11360479; doi:10.1186/s13058-024-01882-x)
Supplement: Supplementary file 6 — Additional file 6. [file 13058_2024_1882_MOESM6_ESM.pdf]

Supplementaty Figure 6

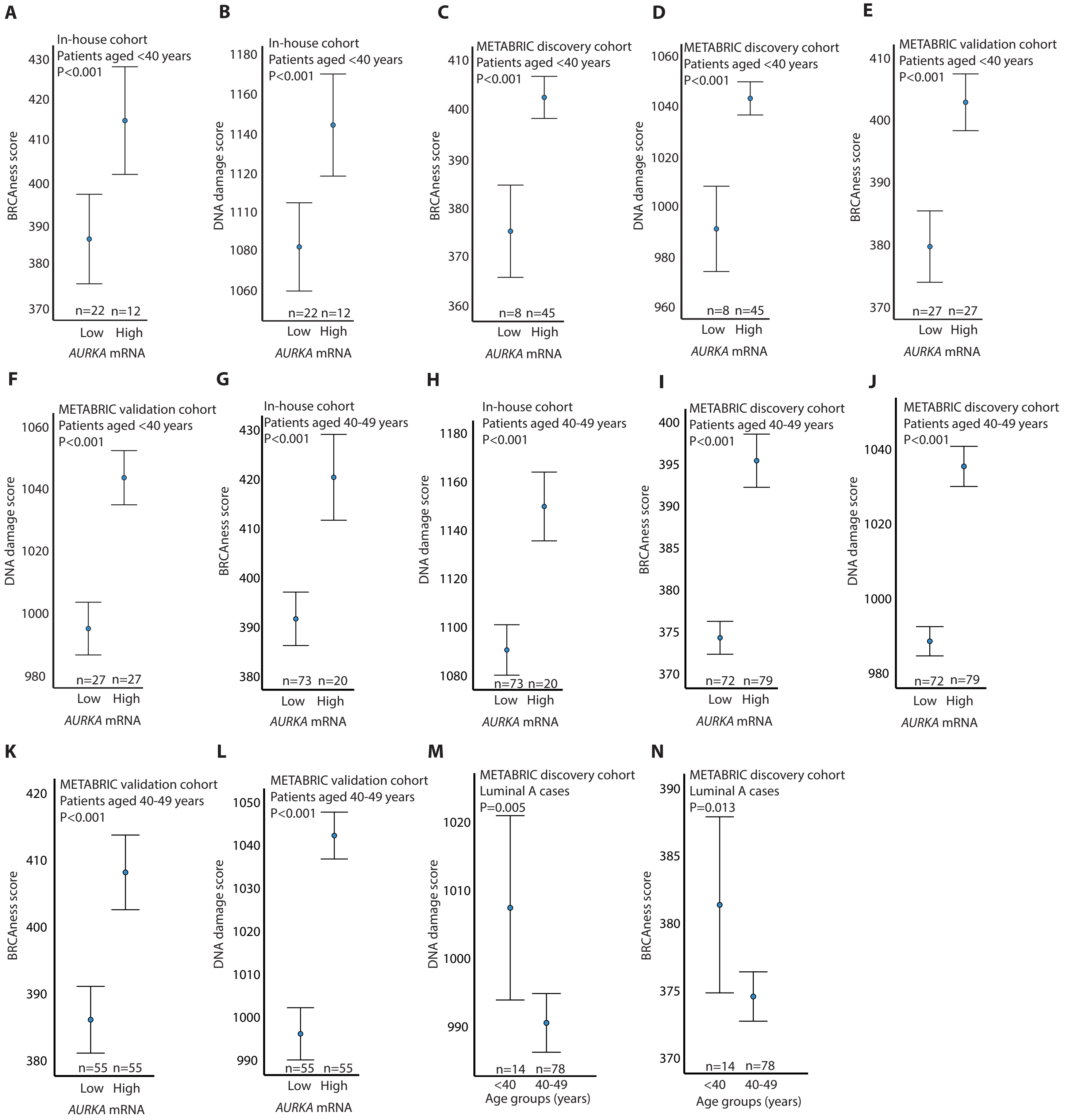

Supplementary Figure 6: **High *AURKA* mRNA associates with increased DNA damage activation and BRCAness score, and high DNAdamage- and BRCAness score associates with young age in luminal A subtype.**
(**A-L**) BRCAness score and DNA damage score calculated from Log2 transformed mRNA across *AURKA* mRNA in patients aged <40 years (A-B; in-house cohort n=34, C-D; METABRIC <50 cohort n=53, E-F; METABRIC <50 validation cohort n=62), and patients aged 40-49 years (G-H; in-house cohort n=93, I-J; METABRIC <50 cohort n=151, K-L;METABRIC <50 validation cohort n=140). (**M-N**) DNA damage- and BRCAness score across *AURKA* mRNA in luminal A cases only (M-N; METABRIC discovery <50 cohort n=92).
