# Supplementary material for: Elevated expression of Aurora-A/AURKA in breast cancer associates with younger age and aggressive features
Source: Breast Cancer Res. 2024 Aug 28;26:126. doi: 10.1186/s13058-024-01882-x (PMC11360479; doi:10.1186/s13058-024-01882-x)
Supplement: Supplementary file 7 — Additional file 7. [file 13058_2024_1882_MOESM7_ESM.pdf]

Supplementary Table 1: Multivariate analysis. Cox' proportional hazards regression with disease-specific death from breast cancer as end-point. <50 METABRIC discovery (n=151) and validation cohort (n=110), patients aged 40-49 only.

**A**	**METABRIC discovery cohort, patients aged 40-49 (n=151)**			
Variables	n	n (%)	Multivariate HR (95% CI)	P-value
Histologic grade				NS
Grade 1 and 2	67	44.4	1	
Grade 3	84	55.6	0.67 (0.29-1.56)	
Tumor diameter				NS
<20mm	70	46.4	1	
>20mm	81	53.6	1.19 (0.60-2.34)	
Nodal status				NS
Negative	82	54.3	1	
Positive	69	45.7	1.54 (0.82-2.88)	
MKI67	151			NS
			1.30 (0.53-3.16)	
AURKA mRNA	151			**<0.001**
			2.25 (1.63-3.10)	

**B**	**METABRIC validation cohort, patients aged 40-49 (n=110)**			
Variables	n	n (%)	Multivariate HR (95% CI)	P-value
Histologic grade*				NS
Grade 1 and 2	53	32.3	1	
Grade 3	106	64.6	2.40 (0.84-6.84)	
Tumor diameter				**0.005**
<20mm	73	44.5	1	
>20mm	91	55.5	3.12 (1.43-7.20)	
Nodal status				NS
Negative	71	43.3	1	
Positive	93	56.7	0.68 (2.95-1.58)	
MKI67	110			NS
			0.69 (0.30-1.61)	
AURKA mRNA	110			**<0.001**
			2.21 (1.50-3.26)	
**Missing: Histologic grade n=5*				

HR=Hazard ratio, CI=Confidence interval, n=number of patients
