# Supplementary material for: Elevated expression of Aurora-A/AURKA in breast cancer associates with younger age and aggressive features
Source: Breast Cancer Res. 2024 Aug 28;26:126. doi: 10.1186/s13058-024-01882-x (PMC11360479; doi:10.1186/s13058-024-01882-x)
Supplement: Supplementary file 8 — Additional file 8. [file 13058_2024_1882_MOESM8_ESM.pdf]

Supplementary Table 2: Differen ally expressed genes (DEGs) in *AURKA* high expression cases from the METABRIC combined cohorts: discovery and valida on datasets with fold change ≥1.5 / ≤ -1.5 and false discovery rate (FDR) <0.008

Upregulated DEGs Gene symbol	AURKA mRNA high, METABRIC cohorts (n=1784)				
	Fold change	FDR	Downregulated DEGs Gene symbol	Fold change	FDR
UBE2C	3.302	<0.008	PIP	4.91	<0.008
S100A8	2.883	<0.008	ANKRD30A	3.58	<0.008
CDC20	2.877	<0.008	AGR3	3.174	<0.008
CBX2	2.637	<0.008	SCUBE2	3.153	<0.008
S100A9	2.617	<0.008	NAT1	3.121	<0.008
SLC7A5	2.595	<0.008	ESR1	2.968	<0.008
CDCA5	2.592	<0.008	SCGB2A2	2.96	<0.008
AURKA	2.582	<0.008	TFF1	2.942	<0.008
C1ORF106	2.478	<0.008	SCGB1D2	2.921	<0.008
CCNB2	2.439	<0.008	LOC644151	2.918	<0.008
PTTG1	2.406	<0.008	LOC646360	2.886	<0.008
MELK	2.365	<0.008	CLIC6	2.698	<0.008
TOP2A	2.363	<0.008	C1ORF64	2.52	<0.008
BIRC5	2.231	<0.008	CYP4X1	2.515	<0.008
CXCL10	2.213	<0.008	TFF3	2.502	<0.008
CXCL9	2.193	<0.008	MAPT	2.428	<0.008
PRC1	2.178	<0.008	DNAJC12	2.405	<0.008
AURKB	2.161	<0.008	STC2	2.372	<0.008
TRIP13	2.136	<0.008	CYP4Z1	2.37	<0.008
CDC45L	2.134	<0.008	NOSTRIN	2.343	<0.008
PTTG3P	2.111	<0.008	CYBRD1	2.337	<0.008
CALML5	2.102	<0.008	NTN4	2.319	<0.008
KIF20A	2.1	<0.008	HS.144479	2.29	<0.008
ASPM	2.09	<0.008	MLPH	2.219	<0.008
TPX2	2.049	<0.008	AZGP1	2.205	<0.008
LOC652694	2.018	<0.008	SUSD3	2.198	<0.008
CDCA7	2.014	<0.008	CA12	2.192	<0.008
E2F2	2.014	<0.008	CX3CR1	2.188	<0.008
RARRES1	2.007	<0.008	CYP4Z2P	2.175	<0.008
FAM83D	1.999	<0.008	LOC338579	2.169	<0.008
LAD1	1.999	<0.008	GATA3	2.131	<0.008
OIP5	1.987	<0.008	LOC388743	2.111	<0.008
UHRF1	1.968	<0.008	BNIPL	2.097	<0.008
MCM10	1.961	<0.008	HS.388347	2.078	<0.008
NCAPG	1.957	<0.008	FCGBP	2.076	<0.008
CENPA	1.945	<0.008	FOXA1	2.069	<0.008
LOC731049	1.945	<0.008	CFB	2.06	<0.008
LOC651816	1.935	<0.008	TBC1D9	2.054	<0.008
PSAT1	1.929	<0.008	SERPINA11	2.024	<0.008
KIF2C	1.926	<0.008	LOC389033	2.003	<0.008
CEP55	1.925	<0.008	FGD3	1.998	<0.008
EXO1	1.92	<0.008	LOC124220	1.992	<0.008
CENPN	1.916	<0.008	HS.389988	1.982	<0.008
TROAP	1.908	<0.008	MUC1	1.975	<0.008

KRT81	1.904	<0.008	AGTR1	1.968	<0.008
CCL8	1.902	<0.008	DACH1	1.963	<0.008
BUB1	1.9	<0.008	HMGCS2	1.955	<0.008
CCNA2	1.891	<0.008	LTF	1.939	<0.008
UBE2T	1.886	<0.008	REEP6	1.939	<0.008
CDC2	1.885	<0.008	HS.570988	1.921	<0.008
MAD2L1	1.884	<0.008	LRRC17	1.918	<0.008
LOC649923	1.876	<0.008	PTPRT	1.911	<0.008
MMP9	1.875	<0.008	PPP1R3C	1.902	<0.008
CDCA3	1.867	<0.008	ENPP5	1.9	<0.008
CCNE1	1.863	<0.008	KIF13B	1.9	<0.008
TTK	1.859	<0.008	GRIA2	1.899	<0.008
KIFC1	1.855	<0.008	TGFBR3	1.892	<0.008
ADAMDEC1	1.852	<0.008	GP2	1.888	<0.008
CDKN3	1.834	<0.008	HS.159264	1.875	<0.008
CENPF	1.833	<0.008	SLC39A6	1.857	<0.008
CDT1	1.832	<0.008	KIF5C	1.844	<0.008
C9ORF140	1.831	<0.008	GRP	1.84	<0.008
TYMS	1.818	<0.008	DNALI1	1.839	<0.008
CDCA8	1.81	<0.008	STK32B	1.839	<0.008
GGH	1.808	<0.008	THBS4	1.834	<0.008
HJURP	1.806	<0.008	CXCL14	1.833	<0.008
FOXM1	1.805	<0.008	SCGB2A1	1.83	<0.008
TK1	1.802	<0.008	MS4A7	1.819	<0.008
TMSB15A	1.792	<0.008	MYB	1.817	<0.008
CDH3	1.78	<0.008	GSTM2	1.812	<0.008
RACGAP1	1.776	<0.008	PSD3	1.808	<0.008
PHGDH	1.772	<0.008	SCNN1A	1.808	<0.008
LAPTM4B	1.769	<0.008	SLC40A1	1.807	<0.008
CDC25B	1.766	<0.008	PDZK1	1.805	<0.008
LCN2	1.756	<0.008	BCL2	1.804	<0.008
CKAP2L	1.75	<0.008	SLC7A2	1.803	<0.008
CKS2	1.75	<0.008	C9ORF152	1.801	<0.008
MCM4	1.743	<0.008	RERG	1.8	<0.008
C6ORF173	1.742	<0.008	HS.7413	1.789	<0.008
KRT80	1.731	<0.008	RNASE4	1.783	<0.008
CENPE	1.73	<0.008	RTN1	1.769	<0.008
RAD51AP1	1.73	<0.008	HS.573062	1.764	<0.008
GZMB	1.725	<0.008	C4ORF18	1.763	<0.008
HS.579631	1.718	<0.008	LRIG1	1.763	<0.008
KYNU	1.717	<0.008	REEP1	1.762	<0.008
C4ORF7	1.712	<0.008	EVL	1.757	<0.008
S100P	1.711	<0.008	TPRG1	1.756	<0.008
NEK2	1.71	<0.008	FMO5	1.753	<0.008
MGC40489	1.708	<0.008	SMOC2	1.751	<0.008
MCM2	1.706	<0.008	SEC14L2	1.749	<0.008
SQLE	1.698	<0.008	SERPINA3	1.748	<0.008
CCNE2	1.697	<0.008	NDP	1.744	<0.008
STIL	1.697	<0.008	GJA1	1.743	<0.008
PLA2G7	1.693	<0.008	CGNL1	1.736	<0.008

FEN1	1.692	<0.008	FOS	1.726	<0.008
STMN1	1.692	<0.008	PGR	1.726	<0.008
KPNA2	1.689	<0.008	COL4A5	1.725	<0.008
POLQ	1.689	<0.008	NME5	1.724	<0.008
DHCR7	1.687	<0.008	QDPR	1.719	<0.008
GBP5	1.682	<0.008	GOLSYN	1.713	<0.008
PFKP	1.681	<0.008	TMEM26	1.712	<0.008
LAMP3	1.678	<0.008	BTG2	1.693	<0.008
HMMR	1.672	<0.008	IRX2	1.692	<0.008
IFI27	1.67	<0.008	GSTM1	1.69	<0.008
LRP8	1.666	<0.008	C10ORF116	1.683	<0.008
MX1	1.665	<0.008	AFF3	1.681	<0.008
ZWINT	1.664	<0.008	LRP2	1.681	<0.008
FAM64A	1.662	<0.008	LYPD6	1.678	<0.008
SOD2	1.661	<0.008	C14ORF45	1.677	<0.008
ATAD2	1.647	<0.008	PLAT	1.675	<0.008
ISG15	1.645	<0.008	SETBP1	1.675	<0.008
CENPM	1.644	<0.008	GSTM3	1.668	<0.008
PITX1	1.642	<0.008	KIAA1324	1.668	<0.008
SOX11	1.637	<0.008	HOXB2	1.667	<0.008
GBP1	1.636	<0.008	CILP	1.66	<0.008
GINS2	1.631	<0.008	SH3BGRL	1.659	<0.008
QPRT	1.629	<0.008	ZNF533	1.648	<0.008
KIF4A	1.627	<0.008	ELOVL5	1.642	<0.008
PKMYT1	1.626	<0.008	CIDEC	1.637	<0.008
INDO	1.622	<0.008	ATP1B1	1.636	<0.008
DLGAP5	1.621	<0.008	PLAC9	1.636	<0.008
HES6	1.619	<0.008	SCGB3A1	1.63	<0.008
TMEM132A	1.618	<0.008	ABCC8	1.626	<0.008
ANLN	1.617	<0.008	PHYHD1	1.624	<0.008
KRT6A	1.617	<0.008	C20ORF103	1.623	<0.008
KIF23	1.608	<0.008	MFAP4	1.614	<0.008
TUBB3	1.601	<0.008	LOC644844	1.612	<0.008
PLK4	1.593	<0.008	MYH11	1.605	<0.008
SPC24	1.586	<0.008	C7ORF41	1.603	<0.008
KIF11	1.581	<0.008	GAMT	1.602	<0.008
EPSTI1	1.578	<0.008	FCER1A	1.595	<0.008
C16ORF61	1.575	<0.008	RGS5	1.592	<0.008
CHEK1	1.573	<0.008	TCN1	1.592	<0.008
CKS1B	1.572	<0.008	MAOA	1.589	<0.008
TAP1	1.572	<0.008	SPARCL1	1.589	<0.008
IMPA2	1.57	<0.008	CIRBP	1.588	<0.008
NDRG1	1.57	<0.008	PKIB	1.585	<0.008
GNLY	1.567	<0.008	C8ORF4	1.584	<0.008
IFI44L	1.567	<0.008	OGN	1.583	<0.008
APOBEC3B	1.561	<0.008	PODN	1.583	<0.008
ECE2	1.561	<0.008	NKX3-1	1.578	<0.008
DSC2	1.56	<0.008	RABEP1	1.575	<0.008
CTSL2	1.559	<0.008	FABP4	1.574	<0.008
KRT86	1.559	<0.008	OMD	1.572	<0.008

GRB7	1.555	<0.008	CASC1	1.57	<0.008
VGLL1	1.555	<0.008	ADH1A	1.566	<0.008
ASF1B	1.553	<0.008	FOSB	1.564	<0.008
MGC39900	1.549	<0.008	C7ORF63	1.561	<0.008
TIMELESS	1.545	<0.008	SLC7A8	1.56	<0.008
GTSE1	1.543	<0.008	COL16A1	1.553	<0.008
C1ORF135	1.538	<0.008	PNPLA7	1.552	<0.008
MMP12	1.538	<0.008	SFRP2	1.552	<0.008
NCCRP1	1.536	<0.008	IGSF21	1.551	<0.008
GSDMB	1.535	<0.008	STEAP2	1.548	<0.008
WDR51A	1.531	<0.008	DCLK1	1.544	<0.008
KIF1A	1.53	<0.008	FST	1.543	<0.008
C17ORF53	1.527	<0.008	C17ORF97	1.537	<0.008
GPSM2	1.526	<0.008	HS.532698	1.535	<0.008
MCM6	1.523	<0.008	TCEAL1	1.535	<0.008
STAT1	1.523	<0.008	KIAA1370	1.534	<0.008
PBK	1.522	<0.008	EPHX2	1.533	<0.008
BOP1	1.52	<0.008	CYB5A	1.529	<0.008
FBXO5	1.517	<0.008	FMOD	1.529	<0.008
LOC731314	1.517	<0.008	ARMCX2	1.524	<0.008
NUSAP1	1.516	<0.008	TGFB3	1.524	<0.008
C16ORF75	1.515	<0.008	SFRP4	1.52	<0.008
SLC16A3	1.515	<0.008	CCL15	1.519	<0.008
MSLN	1.513	<0.008	C1ORF21	1.515	<0.008
EIF2C2	1.512	<0.008	LOC389816	1.51	<0.008
GPR172A	1.512	<0.008	CCDC74A	1.509	<0.008
CBS	1.51	<0.008	MGC18216	1.506	<0.008
CCNB1	1.509	<0.008	SORBS2	1.503	<0.008
HMGA1	1.507	<0.008	HS.400256	1.501	<0.008
ECT2	1.505	<0.008			
MND1	1.505	<0.008			
PRR11	1.504	<0.008			
DDX39	1.502	<0.008			
THOC4	1.502	<0.008			
