# Supplementary material for: Elevated expression of Aurora-A/AURKA in breast cancer associates with younger age and aggressive features
Source: Breast Cancer Res. 2024 Aug 28;26:126. doi: 10.1186/s13058-024-01882-x (PMC11360479; doi:10.1186/s13058-024-01882-x)
Supplement: Supplementary file 9 — Additional file 9. [file 13058_2024_1882_MOESM9_ESM.pdf]

Supplementary Table 3: Gene Sets enriched in *AURKA* mRNA expression high for METABRIC cohorts combined (n=1784), false discovery rate cutoff < 5 %.

Categories/Gene set	False Discovery Rate (%)
**HALLMARKS**	
HALLMARK_E2F_TARGETS	<0.004
HALLMARK_G2M_CHECKPOINT	<0.004
HALLMARK_MTORC1_SIGNALING	0.07
HALLMARK_MYC_TARGETS_V2	0.24
HALLMARK_MYC_TARGETS_V1	0.24
HALLMARK_MITOTIC_SPINDLE	0.24
HALLMARK_UNFOLDED_PROTEIN_RESPONSE	0.48
HALLMARK_DNA_REPAIR	0.5
HALLMARK_UV_RESPONSE_UP	0.73
HALLMARK_SPERMATOGENESIS	0.8
HALLMARK_GLYCOLYSIS	0.98
**Kyoto Encyclopedia of Genes and Genomes (KEGG)**	
KEGG_CELL_CYCLE	<0.004
KEGG_PYRIMIDINE_METABOLISM	0.3
KEGG_PROTEASOME	0.4
KEGG_OOCYTE_MEIOSIS	0.64
KEGG_DNA_REPLICATION	0.78
KEGG_PROGESTERONE_MEDIATED_OOCYTE_MATURATION	0.78
KEGG_PURINE_METABOLISM	2.69
KEGG_BASE_EXCISION_REPAIR	3.27
KEGG_MISMATCH_REPAIR	3.33
KEGG_NON_SMALL_CELL_LUNG_CANCER	4.61
KEGG_ALANINE_ASPARTATE_AND_GLUTAMATE_METABOLISM	4.65
KEGG_HOMOLOGOUS_RECOMBINATION	4.65
KEGG_TERPENOID_BACKBONE_BIOSYNTHESIS	4.79
KEGG_NON_HOMOLOGOUS_END_JOINING	4.87
KEGG_BLADDER_CANCER	4.92
KEGG_NUCLEOTIDE_EXCISION_REPAIR	4.99
**Gene Ontology Biological Process**	
GO_MITOTIC_NUCLEAR_DIVISION	<0.004
GO_MITOTIC_SISTER_CHROMATID_SEGREGATION	0.1
GO_REGULATION_OF_TRANSCRIPTION_INVOLVED_IN_G1_S_TRANSITION_OF_MITOTIC_CELL_CYCLE	0.26
GO_CELL_CYCLE_G1_S_PHASE_TRANSITION	0.27
GO_CHROMOSOME_CONDENSATION	0.27
GO_CHROMOSOME_SEPARATION	0.27
GO_NEGATIVE_REGULATION_OF_CELL_CYCLE_G2_M_PHASE_TRANSITION	0.27
GO_DNA_REPLICATION	0.29
GO_MICROTUBULE_CYTOSKELETON_ORGANIZATION_INVOLVED_IN_MITOSIS	0.29
GO_MITOTIC_SPINDLE_ORGANIZATION	0.29
GO_ORGANELLE_FISSION	0.29
GO_DNA_CONFORMATION_CHANGE	0.3
GO_FEMALE_MEIOTIC_NUCLEAR_DIVISION	0.3

GO_METAPHASE_PLATE_CONGRESSION	0.3
GO_CHROMOSOME_SEGREGATION	0.31
GO_NEGATIVE_REGULATION_OF_CELL_CYCLE_PHASE_TRANSITION	0.31
GO_POSITIVE_REGULATION_OF_MITOTIC_CELL_CYCLE	0.31
GO_MITOTIC_METAPHASE_PLATE_CONGRESSION	0.32
GO_MITOTIC_CELL_CYCLE_CHECKPOINT	0.33
GO_SISTER_CHROMATID_COHESION	0.33
GO_SPINDLE_ORGANIZATION	0.33
GO_DNA_DEPENDENT_DNA_REPLICATION	0.36
GO_METAPHASE_ANAPHASE_TRANSITION_OF_CELL_CYCLE	0.37
GO_CELL_CYCLE_DNA_REPLICATION	0.39
GO_DNA_STRAND_ELONGATION	0.49
GO_NUCLEOSIDE_MONOPHOSPHATE_METABOLIC_PROCESS	0.49
GO_ANAPHASE_PROMOTING_COMPLEX_DEPENDENT_CATABOLIC_PROCESS	0.51
GO_MEIOTIC_CELL_CYCLE_PROCESS	0.51
GO_DNA_PACKAGING	0.56
GO_NEGATIVE_REGULATION_OF_CELL_CYCLE_PROCESS	0.57
GO_DNA_REPLICATION_INITIATION	0.61
GO_CHROMOSOME_LOCALIZATION	0.62
GO_DNA_STRAND_ELONGATION_INVOLVED_IN_DNA_REPLICATION	0.64
GO_CELL_CYCLE_CHECKPOINT	0.65
GO_MEIOTIC_CHROMOSOME_SEGREGATION	0.66
GO_RRNA_METHYLATION	0.9
GO_SPINDLE_ASSEMBLY	0.9
GO_CELL_CYCLE_G2_M_PHASE_TRANSITION	0.91
GO_FOLIC_ACID_CONTAINING_COMPOUND_METABOLIC_PROCESS	1.02
GO_G1_DNA_DAMAGE_CHECKPOINT	1.1
GO_RIBONUCLEOSIDE_MONOPHOSPHATE_BIOSYNTHETIC_PROCESS	1.3
GO_MEIOTIC_CELL_CYCLE	1.38
GO_MEIOSIS_I_CELL_CYCLE_PROCESS	1.62
GO_DOUBLE_STRAND_BREAK_REPAIR	1.8
GO_KINETOCHORE_ORGANIZATION	1.8
GO_POSITIVE_REGULATION_OF_MITOTIC_NUCLEAR_DIVISION	2.11
GO_HISTONE_PHOSPHORYLATION	2.16
GO_DNA_REPAIR	2.25
GO_CHROMOSOME_ORGANIZATION_INVOLVED_IN_MEIOTIC_CELL_CYCLE	2.27
GO_MITOTIC_CHROMOSOME_CONDENSATION	2.27
GO_RIBONUCLEOSIDE_MONOPHOSPHATE_METABOLIC_PROCESS	2.31
GO_TELOMERE_MAINTENANCE_VIA_SEMI_CONSERVATIVE_REPLICATION	2.38
GO_MITOTIC_SPINDLE_ASSEMBLY	2.42
GO_DNA_UNWINDING_INVOLVED_IN_DNA_REPLICATION	2.46
GO_HISTONE_SERINE_PHOSPHORYLATION	2.46
GO_HOMOLOGOUS_CHROMOSOME_SEGREGATION	2.46
GO_REGULATION_OF_SISTER_CHROMATID_SEGREGATION	2.48
GO_DNA_RECOMBINATION	2.5
GO_MITOTIC_DNA_REPLICATION	2.5
GO_CENTROMERE_COMPLEX_ASSEMBLY	2.59
GO_HOMOLOGOUS_RECOMBINATION	2.99
GO_TERMINATION_OF_RNA_POLYMERASE_II_TRANSCRIPTION	3.13
GO_HISTONE_EXCHANGE	3.15

GO_MITOCHONDRIAL_RNA_METABOLIC_PROCESS	3.22
GO_ATTACHMENT_OF_SPINDLE_MICROTUBULES_TO_KINETOCHORE	3.36
GO_CYTOKINETIC_PROCESS	3.53
GO_KINETOCHORE_ASSEMBLY	3.74
GO_CHROMATIN_ASSEMBLY_OR_DISASSEMBLY	3.79
GO_DNA_DEPENDENT_DNA_REPLICATION_MAINTENANCE_OF_FIDELITY	4.07
GO_CHROMATIN_ASSEMBLY	4.08
GO_RNA_MODIFICATION	4.49
GO_NEGATIVE_REGULATION_OF_CELL_CYCLE_G1_S_PHASE_TRANSITION	4.51
GO_POSITIVE_REGULATION_OF_CELL_CYCLE	4.51
GO_ANTIGEN_PROCESSING_AND_PRESENTATION_OF_EXOGENOUS_PEPTIDE_ANTIGEN_V MHC_CLASS_I	4.53
GO_CYTOSKELETON_DEPENDENT_CYTOKINESIS	4.56
GO_DNA_SYNTHESIS_INVOLVED_IN_DNA_REPAIR	4.58
