# Supplementary material for: Elevated expression of Aurora-A/AURKA in breast cancer associates with younger age and aggressive features
Source: Breast Cancer Res. 2024 Aug 28;26:126. doi: 10.1186/s13058-024-01882-x (PMC11360479; doi:10.1186/s13058-024-01882-x)
Supplement: Supplementary file 10 — Additional file 10. [file 13058_2024_1882_MOESM10_ESM.pdf]

Supplementary Table 4: Gene Sets enriched in *AURKA* mRNA expression high for the METABRIC discovery <50 cohort in patients aged below 40 (n = 53), and 40-49 (n=151) years. False discovery rate cutoff <5 %.
Selected gene sets for GO:Biological processes (top 50).

**Patients aged below 40 years (n=58)**	
Categories/Gene set	False Discovery Rate (%)
**HALLMARKS**	
HALLMARK_G2M_CHECKPOINT	<0.008
HALLMARK_E2F_TARGETS	<0.008
HALLMARK_MYC_TARGETS_V2	0.17
HALLMARK_MITOTIC_SPINDLE	0.56
HALLMARK_UNFOLDED_PROTEIN_RESPONSE	0.57
HALLMARK_MTORC1_SIGNALING	0.75
HALLMARK_INTERFERON_ALPHA_RESPONSE	1.44
HALLMARK_DNA_REPAIR	1.72
HALLMARK_INTERFERON_GAMMA_RESPONSE	1.74
HALLMARK_MYC_TARGETS_V1	2.58
HALLMARK_UV_RESPONSE_UP	2.83
**Kyoto Encyclopedia of Genes and Genomes (KEGG)**	
KEGG_CELL_CYCLE	0.11
KEGG_DNA_REPLICATION	0.97
KEGG_HOMOLOGOUS_RECOMBINATION	1.36
KEGG_STEROID_BIOSYNTHESIS	1.51
KEGG_PYRIMIDINE_METABOLISM	1.59
KEGG_OOCYTE_MEIOSIS	1.82
KEGG_PROTEASOME	2.07
KEGG_ONE_CARBON_POOL_BY_FOLATE	2.22
KEGG_BASE_EXCISION_REPAIR	2.31
KEGG_PROGESTERONE_MEDIATED_OOCYTE_MATURATION	2.47
KEGG_MISMATCH_REPAIR	3.24
**Gene Ontology Biological Process**	
GO_MITOTIC_SISTER_CHROMATID_SEGREGATION	<0.008
GO_MITOTIC_NUCLEAR_DIVISION	<0.008
GO_REGULATION_OF_CHROMOSOME_SEGREGATION	<0.008
GO_DNA_DEPENDENT_DNA_REPLICATION	0.02
GO_REGULATION_OF_SISTER_CHROMATID_SEGREGATION	0.02
GO_NUCLEAR_CHROMOSOME_SEGREGATION	0.07
GO_SISTER_CHROMATID_SEGREGATION	0.08
GO_ORGANELLE_FISSION	0.08
GO_METAPHASE_ANAPHASE_TRANSITION_OF_CELL_CYCLE	0.09
GO_REGULATION_OF_NUCLEAR_DIVISION	0.09
GO_CHROMOSOME_SEGREGATION	0.1
GO_DNA_REPLICATION	0.1
GO_NEGATIVE_REGULATION_OF_NUCLEAR_DIVISION	0.1
GO_FEMALE_MEIOTIC_NUCLEAR_DIVISION	0.1
GO_NEGATIVE_REGULATION_OF_CHROMOSOME_SEGREGATION	0.12
GO_REGULATION_OF_CHROMOSOME_SEPARATION	0.15

GO_MITOTIC_SPINDLE_ORGANIZATION	0.18
GO_NEGATIVE_REGULATION_OF_METAPHASE_ANAPHASE_TRANSITION_ OF_CELL_CYCLE	0.19
GO_NEGATIVE_REGULATION_OF_CELL_CYCLE_PHASE_TRANSITION	0.2
GO_CHROMOSOME_CONDENSATION	0.2
GO_CHROMOSOME_SEPARATION	0.2
GO_MITOTIC_METAPHASE_PLATE_CONGRESSION	0.2
GO_CELL_CYCLE_G1_S_PHASE_TRANSITION	0.21
GO_NEGATIVE_REGULATION_OF_CELL_CYCLE_G2_M_PHASE_TRANSITION	0.23
GO_MICROTUBULE_CYTOSKELETON_ORGANIZATION_INVOLVED_IN_MITOSIS	0.3
GO_MEIOTIC_CELL_CYCLE_PROCESS	0.3
GO_PYRIMIDINE_NUCLEOSIDE_METABOLIC_PROCESS	0.31
GO_DNA_CONFORMATION_CHANGE	0.32
GO_POSITIVE_REGULATION_OF_CELL_CYCLE_PHASE_TRANSITION	0.33
GO_SPINDLE_ORGANIZATION	0.35
GO_REGULATION_OF_CELL_CYCLE_PHASE_TRANSITION	0.35
GO_MEIOTIC_SPINDLE_ORGANIZATION	0.35
GO_METAPHASE_PLATE_CONGRESSION	0.35
GO_RECOMBINATIONAL_REPAIR	0.38
GO_BASE_EXCISION_REPAIR	0.39
GO_CELL_CYCLE_DNA_REPLICATION	0.4
GO_MITOTIC_CELL_CYCLE_CHECKPOINT	0.4
GO_DNA_GEOMETRIC_CHANGE	0.44
GO_DNA_STRAND_ELONGATION	0.49
GO_DNA_STRAND_ELONGATION_INVOLVED_IN_DNA_REPLICATION	0.49
GO_NEGATIVE_REGULATION_OF_MITOTIC_CELL_CYCLE	0.5
GO_DNA_PACKAGING	0.6
GO_REGULATION_OF_CELL_CYCLE_G2_M_PHASE_TRANSITION	0.63
GO_REGULATION_OF_MEIOTIC_CELL_CYCLE	0.75
GO_CELL_CYCLE_CHECKPOINT	0.76
GO_NEGATIVE_REGULATION_OF_CHROMOSOME_ORGANIZATION	0.81
GO_CELL_CYCLE_G2_M_PHASE_TRANSITION	0.83
GO_SIGNAL_TRANSDUCTION_INVOLVED_IN_CELL_CYCLE_CHECKPOINT	0.85
GO_NEGATIVE_REGULATION_OF_CELL_CYCLE_PROCESS	0.87

**Patients aged 40-49 years n=151**	
Categories/Gene set	False Discovery Rate (%)
**HALLMARKS**	
HALLMARK_G2M_CHECKPOINT	<0.008
HALLMARK_E2F_TARGETS	<0.008
HALLMARK_MITOTIC_SPINDLE	<0.008
HALLMARK_MTORC1_SIGNALING	<0.008
HALLMARK_UNFOLDED_PROTEIN_RESPONSE	<0.008
HALLMARK_PI3K_AKT_MTOR_SIGNALING	0.02
HALLMARK_MYC_TARGETS_V2	0.07
HALLMARK_MYC_TARGETS_V1	0.1
HALLMARK_UV_RESPONSE_UP	0.15
HALLMARK_SPERMATOGENESIS	0.27
HALLMARK_CHOLESTEROL_HOMEOSTASIS	0.89

HALLMARK_REACTIVE_OXYGEN_SPECIES_PATHWAY	0.98
HALLMARK_DNA_REPAIR	1.14
HALLMARK_ALLOGRAFT_REJECTION	1.52
HALLMARK_GLYCOLYSIS	1.71
HALLMARK_INTERFERON_GAMMA_RESPONSE	1.62
HALLMARK_INTERFERON_ALPHA_RESPONSE	2.87
HALLMARK_COMPLEMENT	3.7
HALLMARK_INFLAMMATORY_RESPONSE	4.46
**Kyoto Encyclopedia of Genes and Genomes (KEGG)**	
KEGG_CELL_CYCLE	<0.008
KEGG_OOCYTE_MEIOSIS	<0.008
KEGG_PROGESTERONE_MEDIATED_OOCYTE_MATURATION	<0.008
KEGG_PROTEASOME	<0.008
KEGG_BLADDER_CANCER	<0.008
KEGG_DNA_REPLICATION	0.09
KEGG_DORSO_VENTRAL_AXIS_FORMATION	0.82
KEGG_HOMOLOGOUS_RECOMBINATION	0.84
KEGG_PYRIMIDINE_METABOLISM	1.35
KEGG_NATURAL_KILLER_CELL_MEDIATED_CYTOTOXICITY	1.55
KEGG_THYROID_CANCER	1.63
KEGG_T_CELL_RECEPTOR_SIGNALING_PATHWAY	1.75
KEGG_ONE_CARBON_POOL_BY_FOLATE	1.75
KEGG_SPLICEOSOME	1.77
KEGG_NON_SMALL_CELL_LUNG_CANCER	1.85
KEGG_GLIOMA	1.92
KEGG_GAP_JUNCTION	2.07
KEGG_CYSTEINE_AND_METHIONINE_METABOLISM	2.14
KEGG_STEROID_BIOSYNTHESIS	2.16
KEGG_RENAL_CELL_CARCINOMA	2.25
KEGG_ERBB_SIGNALING_PATHWAY	2.29
KEGG_FC_GAMMA_R_MEDIATED_PHAGOCYTOSIS	2.33
KEGG_PENTOSE_PHOSPHATE_PATHWAY	2.38
KEGG_RNA_DEGRADATION	2.49
KEGG_ALLOGRAFT_REJECTION	2.57
KEGG_PATHOGENIC_ESCHERICHIA_COLI_INFECTION	2.68
KEGG_NOD_LIKE_RECEPTOR_SIGNALING_PATHWAY	2.89
KEGG_P53_SIGNALING_PATHWAY	2.9
KEGG_MISMATCH_REPAIR	2.95
KEGG_ANTIGEN_PROCESSING_AND_PRESENTATION	3.93
KEGG_TYPE_I_DIABETES_MELLITUS	3.94
KEGG_GALACTOSE_METABOLISM	3.97
KEGG_PRIMARY_IMMUNODEFICIENCY	3.97
KEGG_VIBRIO_CHOLERAE_INFECTION	3.98
KEGG_PROSTATE_CANCER	4.03
KEGG_PURINE_METABOLISM	4.06
KEGG_AUTOIMMUNE_THYROID_DISEASE	4.35
KEGG_NEUROTROPHIN_SIGNALING_PATHWAY	4.46
KEGG_GRAFT_VERSUS_HOST_DISEASE	4.77
KEGG_CHEMOKINE_SIGNALING_PATHWAY	4.81

KEGG_ADHERENS_JUNCTION	4.82
**Gene Ontology Biological Process**	
GO_REGULATION_OF_NUCLEAR_DIVISION	<0.008
GO_ORGANELLE_FISSION	<0.008
GO_MITOTIC_NUCLEAR_DIVISION	<0.008
GO_CHROMOSOME_SEGREGATION	<0.008
GO_NUCLEAR_CHROMOSOME_SEGREGATION	<0.008
GO_MITOTIC_SISTER_CHROMATID_SEGREGATION	<0.008
GO_CYTOKINESIS	<0.008
GO_SISTER_CHROMATID_SEGREGATION	<0.008
GO_CELL_CYCLE_G2_M_PHASE_TRANSITION	<0.008
GO_MEIOTIC_CELL_CYCLE_PROCESS	<0.008
GO_REGULATION_OF_CYTOKINESIS	<0.008
GO_REGULATION_OF_CELL_CYCLE_G2_M_PHASE_TRANSITION	<0.008
GO_REGULATION_OF_CELL_CYCLE_PHASE_TRANSITION	<0.008
GO_REGULATION_OF_CHROMOSOME_SEGREGATION	<0.008
GO_MEIOTIC_CELL_CYCLE	<0.008
GO_NEGATIVE_REGULATION_OF_CELL_CYCLE_PROCESS	<0.008
GO_SPINDLE_ORGANIZATION	<0.008
GO_POSITIVE_REGULATION_OF_CELL_CYCLE_PROCESS	<0.008
GO_CYTOSKELETON_DEPENDENT_CYTOKINESIS	<0.008
GO_POSITIVE_REGULATION_OF_CELL_CYCLE_PHASE_TRANSITION	<0.008
GO_NEGATIVE_REGULATION_OF_CELL_CYCLE_PHASE_TRANSITION	<0.008
GO_POSITIVE_REGULATION_OF_MITOTIC_CELL_CYCLE	<0.008
GO_SPINDLE_ASSEMBLY	<0.008
GO_REGULATION_OF_CYCLIN_DEPENDENT_PROTEIN_KINASE_ACTIVITY	<0.008
GO_ANAPHASE_PROMOTING_COMPLEX_DEPENDENT_CATABOLIC_PROCESS	<0.008
GO_NEGATIVE_REGULATION_OF_MITOTIC_CELL_CYCLE	<0.008
GO_NEGATIVE_REGULATION_OF_CHROMOSOME_ORGANIZATION	<0.008
GO_CHROMOSOME_SEPARATION	<0.008
GO_DNA_REPLICATION	<0.008
GO_REGULATION_OF_SISTER_CHROMATID_SEGREGATION	<0.008
GO_POSITIVE_REGULATION_OF_CELL_CYCLE	<0.008
GO_DNA_DEPENDENT_DNA_REPLICATION	<0.008
GO_CELL_CYCLE_G1_S_PHASE_TRANSITION	<0.008
GO_DNA_CONFORMATION_CHANGE	<0.008
GO_CYTOKINETIC_PROCESS	<0.008
GO_CHROMOSOME_LOCALIZATION	<0.008
GO_NEGATIVE_REGULATION_OF_NUCLEAR_DIVISION	<0.008
GO_DNA_PACKAGING	<0.008
GO_METAPHASE_PLATE_CONGRESSION	<0.008
GO_POSITIVE_REGULATION_OF_MITOTIC_NUCLEAR_DIVISION	<0.008
GO_REGULATION_OF_CHROMOSOME_SEPARATION	<0.008
GO_MITOTIC_METAPHASE_PLATE_CONGRESSION	<0.008
GO_MITOTIC_SPINDLE_ORGANIZATION	<0.008
GO_MITOTIC_CYTOKINESIS	<0.008
GO_MEIOSIS_I_CELL_CYCLE_PROCESS	<0.008
GO_MITOTIC_CELL_CYCLE_CHECKPOINT	<0.008
GO_REGULATION_OF_CENTROSOME_CYCLE	<0.008

GO_FEMALE_MEIOTIC_NUCLEAR_DIVISION	<0.008
GO_METAPHASE_ANAPHASE_TRANSITION_OF_CELL_CYCLE	<0.008
GO_NEGATIVE_REGULATION_OF_CELL_CYCLE_G2_M_PHASE_TRANSITION	<0.008
