# Supplementary material for: Elevated expression of Aurora-A/AURKA in breast cancer associates with younger age and aggressive features
Source: Breast Cancer Res. 2024 Aug 28;26:126. doi: 10.1186/s13058-024-01882-x (PMC11360479; doi:10.1186/s13058-024-01882-x)
Supplement: Supplementary file 11 — Additional file 11. [file 13058_2024_1882_MOESM11_ESM.pdf]

Supplementary Table 5: Uniquely differentially expressed genes (DEGs) between *AURKA* mRNA expression high and low in patients aged <40 and ≥40 years from the combined METABRIC discovery and validation <50 cohorts, n=368
Fold change ≥1.5 / ≤ -1.5, false discovery rate (FDR) <0.008%

Unique upregulated DEGs					
AURKA mRNA high					
<40 years	FDR	Fold change	40-49 years	FDR	Fold change
GABRP	7.813	2.647	S100A8	<0.008	2.853
CTSL2	<0.008	2.203	S100A9	<0.008	2.521
BOP1	<0.008	2.106	CALML5	<0.008	2.067
CTPS	<0.008	1.957	RARRES1	<0.008	1.905
PIR	<0.008	1.876	MMP9	<0.008	1.903
ATP6V0A4	<0.008	1.836	LOC649923	<0.008	1.882
EIF2C2	0.49	1.806	MAD2L1	<0.008	1.858
EZH2	<0.008	1.802	KRT81	<0.008	1.794
DDX39	<0.008	1.787	LAPTM4B	<0.008	1.754
CDH3	7.424	1.777	HS.579631	<0.008	1.733
AIF1L	0.579	1.775	KRT80	<0.008	1.722
HMGA1	<0.008	1.77	CKS2	<0.008	1.713
CEBPB	<0.008	1.76	S100P	<0.008	1.712
NOP2	0.137	1.759	TMSB15A	<0.008	1.708
NCAPD2	0.225	1.757	KYNU	<0.008	1.703
C11ORF82	<0.008	1.75	NEK2	<0.008	1.703
CDCA2	<0.008	1.75	SQLE	<0.008	1.687
LMNB2	<0.008	1.744	GZMB	<0.008	1.679
RFC4	<0.008	1.742	PLA2G7	<0.008	1.669
CLCC1	<0.008	1.738	IFI27	<0.008	1.665
UCK2	<0.008	1.738	LCN2	<0.008	1.664
LOC728564	<0.008	1.733	PFKP	<0.008	1.647
NME1	0.14	1.723	PITX1	<0.008	1.645
KIF14	<0.008	1.717	ATAD2	<0.008	1.633
MCM6	<0.008	1.699	HES6	<0.008	1.628
PUF60	<0.008	1.685	MX1	<0.008	1.617
MMP7	1.419	1.682	GBP1	<0.008	1.609
CD38	<0.008	1.68	SOX11	<0.008	1.606
GPSM2	0.218	1.668	QPRT	<0.008	1.604
RNASEH2A	<0.008	1.666	GINS2	<0.008	1.596
PTDSS1	<0.008	1.657	GRB7	<0.008	1.587
SKP2	0.222	1.657	TUBB3	<0.008	1.582
RECQL4	<0.008	1.654	TMEM132A	<0.008	1.581
PSMG1	<0.008	1.65	ALG1L	<0.008	1.566
PRR11	<0.008	1.648	EPSTI1	<0.008	1.548
PSMB9	4.848	1.635	NDRG1	<0.008	1.545
PGAM5	<0.008	1.627	GSDMB	<0.008	1.541
BCL11A	0.972	1.625	TIMELESS	<0.008	1.541
C13ORF34	0.153	1.62	GNLY	<0.008	1.536
GBP4	5.649	1.619	APOBEC3B	<0.008	1.533
WDR67	<0.008	1.619	WDR51A	<0.008	1.528
MASTL	<0.008	1.612	IFI44L	<0.008	1.524

DONSON	<0.008	1.606	STAT1	<0.008	1.52
MTERFD1	<0.008	1.606	IMPA2	<0.008	1.515
C16ORF59	<0.008	1.604	MMP12	<0.008	1.515
C9ORF58	<0.008	1.598	FBXO5	<0.008	1.512
BLM	<0.008	1.593	SLC16A3	<0.008	1.51
SKA1	<0.008	1.592	KRT86	<0.008	1.509
PRDX4	<0.008	1.59	CCNB1	<0.008	1.508
TMEM189	<0.008	1.58	GPR172A	<0.008	1.502
MCM3	<0.008	1.576	KIF1A	<0.008	1.5
CHD7	<0.008	1.574			
WARS	3.591	1.573			
RCOR2	2.256	1.572			
CENPL	<0.008	1.568			
RHBDF2	0.539	1.568			
MCM7	<0.008	1.553			
STIP1	<0.008	1.553			
LAG3	3.329	1.552			
CD24	<0.008	1.551			
MRPL12	<0.008	1.549			
HAPLN3	4.049	1.546			
KIF15	0.287	1.545			
POLA2	<0.008	1.545			
E2F3	0.234	1.541			
GPT2	6.309	1.538			
SLC25A19	0.373	1.529			
DEPDC1	<0.008	1.528			
TACC3	0.223	1.526			
TFAP2C	4.081	1.525			
ASNS	1.623	1.516			
DKC1	<0.008	1.511			
MND1	<0.008	1.51			
KCNK1	9.647	1.509			
MRPS12	<0.008	1.504			

**Unique downregulated DEGs**					
***AURKA* mRNA low**					
<40 years	FDR	Fold change	≥40 years	FDR	Fold change
CYP4F22	<0.008	5.208	ANKRD30A	<0.008	3.245
TPSAB1	1.318	4.046	TFF1	<0.008	2.783
PRSS23	<0.008	3.9	SCGB1D2	<0.008	2.779
AGR2	0.919	3.724	SCGB2A2	<0.008	2.722
CPA3	1.832	3.074	CLIC6	<0.008	2.627
XBP1	1.298	2.858	C1ORF64	<0.008	2.372
CCDC74A	3.438	2.733	TFF3	<0.008	2.288
DCLK1	0.541	2.706	AZGP1	<0.008	2.106
TPSG1	0.972	2.641	LOC338579	<0.008	2.092
KIAA1370	<0.008	2.624	BNIPL	<0.008	2.073
POSTN	2.075	2.527	FCGBP	<0.008	2.064
FLJ22184	1.556	2.441	MLPH	<0.008	2.054
CTSG	3.696	2.44	SERPINA11	<0.008	1.968

RHOB	0.555	2.431	LTF	<0.008	1.964
SYTL2	2.563	2.415	CFB	<0.008	1.963
ME3	<0.008	2.337	HS.388347	<0.008	1.955
NFIA	0.518	2.284	MUC1	<0.008	1.917
TCEAL3	0.98	2.278	LOC124220	<0.008	1.908
TCEAL1	0.726	2.242	TGFBR3	<0.008	1.908
CPE	2.052	2.239	GP2	<0.008	1.848
CAMK2B	2.271	2.226	ENPP5	<0.008	1.846
CTSK	1.419	2.219	THBS4	<0.008	1.839
CCNG2	<0.008	2.218	GSTM3	<0.008	1.812
UGDH	1.925	2.203	SLC39A6	<0.008	1.802
AMPH	4.797	2.176	KIF5C	<0.008	1.797
MGC18216	4.88	2.09	GSTM1	<0.008	1.785
TBX3	1.459	2.083	CXCL14	<0.008	1.778
LAMB2	0.548	2.065	PSD3	<0.008	1.778
SLC7A8	1.314	2.059	SCGB2A1	<0.008	1.774
ECM2	0.9	2.042	BCL2	<0.008	1.769
HS.532698	<0.008	2.042	HS.159264	<0.008	1.762
HTRA1	2.587	1.997	SERPINA3	<0.008	1.754
TCEAL4	0.758	1.995	RERG	<0.008	1.74
SPARC	3.134	1.98	SLC7A2	<0.008	1.719
COLEC12	3.016	1.979	MYB	<0.008	1.718
C4ORF34	4.983	1.949	CGNL1	<0.008	1.717
DUSP6	2.568	1.944	IRX2	<0.008	1.716
COL4A6	<0.008	1.93	FMO5	<0.008	1.715
CCNDBP1	<0.008	1.927	TPRG1	<0.008	1.711
ITPR1	4.929	1.878	EVL	<0.008	1.703
AHNAK	0.459	1.866	NDP	<0.008	1.703
FRMD6	3.417	1.858	REEP1	<0.008	1.699
MGC42367	2.22	1.853	GSTM2	<0.008	1.687
ITGB5	4.934	1.83	LRP2	<0.008	1.676
FUT8	2.043	1.815	QDPR	<0.008	1.671
PRICKLE2	0.857	1.814	SCGB3A1	<0.008	1.661
GREB1	3.981	1.808	HMGCS2	<0.008	1.66
MEIS3P1	1.191	1.808	LYPD6	<0.008	1.652
CST3	4.994	1.799	BTG2	<0.008	1.646
MYO5C	3.848	1.795	MYH11	<0.008	1.642
TMEM91	1.275	1.793	CILP	<0.008	1.641
C4ORF32	2.234	1.777	KIAA1324	<0.008	1.631
LZTFL1	0.456	1.756	ATP1B1	<0.008	1.626
ECHDC2	1.759	1.746	CIDEC	<0.008	1.622
GALNT10	2.012	1.723	AFF3	<0.008	1.612
HIGD1A	4.374	1.702	ZNF533	<0.008	1.607
MAP3K1	3.489	1.65	PHYHD1	<0.008	1.606
LHFP	3.749	1.633	MFAP4	<0.008	1.603
OLFML3	2.048	1.631	HOXB2	<0.008	1.602
GLI3	2.548	1.628	SH3BGRL	<0.008	1.602
MZF1	1.863	1.627	FCER1A	<0.008	1.601
LIMA1	2.52	1.62	C10ORF116	<0.008	1.599
ZBTB4	<0.008	1.617	C20ORF103	<0.008	1.594

PTN	3.734	1.609	FOSB	<0.008	1.594
C1ORF21	3.723	1.602	TCN1	<0.008	1.586
MXD4	1.997	1.602	ADH1A	<0.008	1.576
SH3D19	1.121	1.6	KIAA1881	<0.008	1.576
RECK	0.508	1.581	LOC644844	<0.008	1.576
CYP21A2	4.819	1.554	MAOA	<0.008	1.576
ZCCHC24	4.391	1.54	GRIA2	<0.008	1.566
HS.13291	4.972	1.539	NKX3-1	<0.008	1.559
APPL2	2.08	1.518	FST	<0.008	1.556
			GAMT	<0.008	1.548
			FABP4	<0.008	1.546
			COL16A1	<0.008	1.544
			SORBS2	<0.008	1.54
			EPHX2	<0.008	1.539
			CCL15	<0.008	1.538
			PKIB	<0.008	1.53
			FMOD	<0.008	1.525
			SFRP2	<0.008	1.522
			C17ORF97	<0.008	1.518
			FRZB	<0.008	1.515
			ARMCX2	<0.008	1.514
			PNPLA7	<0.008	1.511
			IGSF21	<0.008	1.505
