# Supplementary material for: Elevated expression of Aurora-A/AURKA in breast cancer associates with younger age and aggressive features
Source: Breast Cancer Res. 2024 Aug 28;26:126. doi: 10.1186/s13058-024-01882-x (PMC11360479; doi:10.1186/s13058-024-01882-x)
Supplement: Supplementary file 12 — Additional file 12. [file 13058_2024_1882_MOESM12_ESM.pdf]

Supplementary Table 6: Pathway enrichment analysis from uniquely differentially expressed genes (DEGs) in *AURKA* mRNA high between breast cancer patients aged <40 and ≥40 years from the combined METABRIC discovery and validation <50 cohorts, n=368 using the g:Profiler g:GOSt Functional multiquery.

	AURKA **mRNA expression high - uniquely upregulated DEGs**			
			**<40 years**	**≥40years**
**Gene set**	**Category**	**Term ID**	**adjusted P-value**	
GO:BP	DNA unwindings involved in DNA replication	GO:0006268	7.83E-08	1
GO:BP	DNA-templated DNA replication	GO:0006261	2.672E-06	1
GO:BP	DNA duplex unwinding	GO:0032508	7.323E-06	1
GO:BP	chromosome organization	GO:0051276	9.008E-06	1
GO:BP	DNA geometric change	GO:0032392	1.229E-05	1
GO:BP	mitotic cell cycle	GO:0000278	1.872E-05	1
GO:BP	DNA conformation change	GO:0071103	2.315E-05	1
GO:BP	DNA replication	GO:0006260	2.364E-05	1
GO:BP	mitotic cell cycle process	GO:1903047	9.672E-05	1
GO:BP	defense response to other organism	GO:0098542	1	0.000308803
GO:BP	cell cycle	GO:0007049	0.0004172	1
GO:BP	regulation of DNA-templated DNA replication	GO:0090329	0.0008228	0.324962886
GO:BP	defense response	GO:0006952	1	0.000951799
GO:BP	cell cycle process	GO:0022402	0.0016811	1
GO:BP	DNA metabolic process	GO:0006259	0.0041334	1
GO:BP	response to other organism	GO:0051707	1	0.005577983
GO:BP	response to external biotic stimulus	GO:0043207	1	0.00570365
GO:BP	defense response to virus	GO:0051607	1	0.006440187
GO:BP	defense response to symbiont	GO:0140546	1	0.006580528
GO:BP	response to biotic stimulus	GO:0009607	1	0.007476298
GO:BP	neutrophil aggregation	GO:0070488	1	0.008228403
GO:BP	nuclear DNA replication	GO:0033260	0.0093709	1
GO:BP	DNA replication initiation	GO:0006270	0.0093709	1
GO:BP	regulation of cell cycle	GO:0051726	0.0101178	1
GO:BP	double-strand break repair via break-induced replication	GO:0000727	0.0102392	1
GO:BP	innate immune response	GO:0045087	1	0.01047095
GO:BP	cell cycle DNA replication	GO:0044786	0.01549	1
GO:BP	biological process involved in interspecies interaction between organisms	GO:0044419	1	0.018400218
GO:BP	regulation of DNA-templated DNA replication initiation	GO:0030174	0.0210393	1
GO:BP	DNA strand elongation involved in DNA replication	GO:0006271	0.0210393	1
GO:BP	cell population proliferation	GO:0008283	0.0307048	1
GO:BP	immune system process	GO:0002376	1	0.038247286
GO:BP	response to virus	GO:0009615	1	0.039430115
GO:BP	sequestering of metal ion	GO:0051238	1	0.042111636
GO:BP	organelle organization	GO:0006996	0.043821	0.428395465
GO:BP	negative regulation of epithelial cell differentiation involved in kidney development	GO:2000697	1	0.049236417
KEGG	DNA replication	KEGG:03030	3.63E-07	1
KEGG	IL-17 signaling pathway	KEGG:04657	1	0.008192581
KEGG	Cell cycle	KEGG:04110	0.0284997	0.664353598

KEGG	Oocyte meiosis	KEGG:04114	1	0.031316736
REAC	Cell Cycle	REAC:R-HSA-1640170	4.816E-05	1
REAC	DNA strand elongation	REAC:R-HSA-69190	9.731E-05	1
REAC	Metal sequestration by antimicrobial proteins	REAC:R-HSA-6799990	1	0.000259571
REAC	Synthesis of DNA	REAC:R-HSA-69239	0.0003543	1
REAC	Cell Cycle, Mitotic	REAC:R-HSA-69278	0.0012992	1
REAC	Mitotic G1 phase and G1/S transition	REAC:R-HSA-453279	0.0013095	1
REAC	S Phase	REAC:R-HSA-69242	0.0024923	1
REAC	Activation of the pre-replicative complex	REAC:R-HSA-68962	0.0048237	1
REAC	Orc1 removal from chromatin	REAC:R-HSA-68949	0.0055264	1
REAC	DNA Replication	REAC:R-HSA-69306	0.0065961	1
REAC	Unwinding of DNA	REAC:R-HSA-176974	0.0066444	1
REAC	Activation of ATR in response to replication stress	REAC:R-HSA-176187	0.0076846	1
REAC	G1/S Transition	REAC:R-HSA-69206	0.0083013	1
REAC	Cell Cycle Checkpoints	REAC:R-HSA-69620	0.0134346	1
REAC	Switching of origins to a post-replicative state	REAC:R-HSA-69052	0.0194667	1
REAC	Extension of Telomeres	REAC:R-HSA-180786	0.0277793	1
REAC	G2/M Checkpoints	REAC:R-HSA-69481	0.0338368	1

	***AURKA* mRNA expression high - uniquely downregulated DEGs**		**<40 years**	**≥40years**
**Gene set**	**Category**	**Term ID**	**adjusted p value**	
GO:BP	nitrobenzene metabolic process	GO:0018916	1	0.000238167
GO:BP	extracellular matrix organization	GO:0030198	0.0003696	1
GO:BP	extracellular structure organization	GO:0043062	0.0003794	1
GO:BP	external encapsulating structure organization	GO:0045229	0.0003996	1
GO:BP	cellular detoxification of nitrogen compound	GO:0070458	1	0.000594022
GO:BP	detoxification of nitrogen compound	GO:0051410	1	0.000594022
GO:BP	response to xenobiotic stimulus	GO:0009410	1	0.002501256
GO:BP	response to endogenous stimulus	GO:0009719	1	0.002913282
GO:BP	benzene-containing compound metabolic process	GO:0042537	1	0.003601559
GO:BP	anatomical structure morphogenesis	GO:0009653	0.0057256	1
GO:BP	response to hormone	GO:0009725	1	0.007823664
GO:BP	response to stimulus	GO:0050896	1	0.013127772
GO:BP	extracellular matrix disassembly	GO:0022617	0.0450197	1
GO:CC	extracellular space	GO:0005615	0.0002038	2.26E-11
GO:CC	extracellular region	GO:0005576	0.0011005	9.94E-10
GO:CC	extracellular exosome	GO:0070062	0.3195769	0.000104379

GO:CC	extracellular vesicle	GO:1903561	0.1120731	0.000126829
GO:CC	extracellular membrane-bounded organelle	GO:0065010	0.1126354	0.000127855
GO:CC	extracellular organelle	GO:0043230	0.1126354	0.000127855
GO:CC	collagen-containing extracellular matrix	GO:0062023	0.0005657	0.0025123
GO:CC	extracellular matrix	GO:0031012	0.0006799	0.003444067
GO:CC	external encapsulating structure	GO:0030312	0.0006907	0.00349691
GO:CC	vesicle	GO:0031982	0.0440407	0.116805937
KEGG	Drug metabolism - cytochrome P450	KEGG:00982	1	5.43101E-05
KEGG	Platinum drug resistance	KEGG:01524	1	0.028034775
KEGG	Metabolism of xenobiotics by cytochrome P450	KEGG:00980	1	0.031140635
REAC	Extracellular matrix organization	REAC:R-HSA-1474244	0.0015255	1
REAC	Degradation of the extracellular matrix	REAC:R-HSA-1474228	0.0307149	1
REAC	ECM proteoglycans	REAC:R-HSA-3000178	0.035251	1

GO:BP = Gene Ontology Biological Process; KEGG = Kyoto Encyclopedia of Genes and Genomes;
REAC = Reactome; DEGs = Differentially Expressed Genes
