# Supplementary material for: Elevated expression of Aurora-A/AURKA in breast cancer associates with younger age and aggressive features
Source: Breast Cancer Res. 2024 Aug 28;26:126. doi: 10.1186/s13058-024-01882-x (PMC11360479; doi:10.1186/s13058-024-01882-x)
Supplement: Supplementary file 13 — Additional file 13. [file 13058_2024_1882_MOESM13_ESM.pdf]

Supplementary Table 7: Gene Sets enriched in *AURKA* mRNA expression high in the detected uniquely expressed DEGs in young patients using the Cytoscape app BiNGO showing overrepresented GO categories adjusted for multiple testing by the Benjamini Hochberg False Discovery Rate (FDR) correction method. Combined METABRIC discovery and validation <50 cohorts, n=368.

GO:BP term	P-value
DNA strand elongation	1.32E-07
DNA strand elongation involved in DNA replication	1.32E-07
S phase of mitotic cell cycle	7.61E-07
S phase	9.51E-07
pre-replicative complex assembly	1.17E-06
DNA-dependent DNA replication	5.15E-06
double-strand break repair via break-induced replication	5.39E-06
DNA-dependent DNA replication initiation	6.78E-06
double-strand break repair via homologous recombination	2.35E-05
DNA replication	3.11E-05
recombinational repair	4.24E-05
DNA unwinding involved in replication	1.02E-04
DNA recombination	1.09E-04
protein-DNA complex assembly	1.25E-04
DNA duplex unwinding	1.45E-04
DNA geometric change	1.94E-04
double-strand break repair	2.10E-04
DNA metabolic process	2.14E-04
interphase of mitotic cell cycle	2.56E-04
interphase	2.99E-04
chromatin silencing	2.99E-04
negative regulation of gene expression, epigenetic	2.99E-04
DNA repair	3.45E-04
gene silencing	3.57E-04
cell cycle process	3.73E-04
regulation of gene expression, epigenetic	4.55E-04
response to DNA damage stimulus	6.37E-04
cell cycle	7.99E-04
nucleic acid metabolic process	8.88E-04
chromatin silencing at silent mating-type cassette	9.64E-04
negative regulation of transcription, DNA-dependent	1.74E-03
negative regulation of RNA metabolic process	1.77E-03
cell cycle phase	1.86E-03
nucleobase, nucleoside, nucleotide and nucleic acid metabolic process	2.32E-03
negative regulation of transcription	2.40E-03
negative regulation of gene expression	2.54E-03
chromatin silencing at telomere	2.96E-03
negative regulation of nucleobase, nucleoside, nucleotide and nucleic acid metabolic process	3.40E-03
negative regulation of nitrogen compound metabolic process	3.40E-03

cellular response to stress	3.45E-03
negative regulation of macromolecule biosynthetic process	3.57E-03
negative regulation of cellular biosynthetic process	4.13E-03
negative regulation of biosynthetic process	4.22E-03
DNA conformation change	4.29E-03
cellular component biogenesis	4.88E-03
cellular nitrogen compound metabolic process	5.20E-03
negative regulation of cellular metabolic process	5.28E-03
negative regulation of macromolecule metabolic process	5.98E-03
nitrogen compound metabolic process	6.11E-03
cellular response to stimulus	6.98E-03
negative regulation of metabolic process	7.34E-03
mitotic cell cycle	7.41E-03
cellular macromolecular complex assembly	9.35E-03
primary metabolic process	9.41E-03
positive regulation of cell cycle	1.01E-02
negative regulation of cellular process	1.13E-02
cellular metabolic process	1.28E-02
response to stress	1.29E-02
negative regulation of biological process	1.49E-02
cellular macromolecule metabolic process	1.57E-02
macromolecular complex assembly	1.76E-02
macromolecule metabolic process	1.88E-02
metabolic process	1.95E-02
leading strand elongation	2.01E-02

GO:BP = Gene Ontology Biological Process
DEGs = differentially expressed genes
