# Supplementary material for: Elevated expression of Aurora-A/AURKA in breast cancer associates with younger age and aggressive features
Source: Breast Cancer Res. 2024 Aug 28;26:126. doi: 10.1186/s13058-024-01882-x (PMC11360479; doi:10.1186/s13058-024-01882-x)
Supplement: Supplementary file 14 — Additional file 14. [file 13058_2024_1882_MOESM14_ESM.pdf]

Supplementary Table 8: Gene sets enriched in Ki67 expression high for METABRIC cohorts combined (n=1784), false discovery rate cutoff <5%.
For Gene ontology biological processes: top 50 enriched gene sets are represented

Categories/Gene set	False Discovery Rate (%)
HALLMARKS	
HALLMARK_MITOTIC_SPINDLE	0
HALLMARK_G2M_CHECKPOINT	0
HALLMARK_MYC_TARGETS_V2	0.02
HALLMARK_E2F_TARGETS	0.03
HALLMARK_UNFOLDED_PROTEIN_RESPONSE	0.12
HALLMARK_PI3K_AKT_MTOR_SIGNALING	0.73
HALLMARK_SPERMATOGENESIS	0.82
HALLMARK_MTORC1_SIGNALING	2.26
HALLMARK_WNT_BETA_CATENIN_SIGNALING	2.35
HALLMARK_UV_RESPONSE_UP	2.4
HALLMARK_DNA_REPAIR	2.83
HALLMARK_MYC_TARGETS_V1	2.89
Kyoto Encyclopedia of Genes and Genomes (KEGG)	
KEGG_PROGESTERONE_MEDIATED_OOCYTE_MATURATION	0.26
KEGG_DNA_REPLICATION	0.27
KEGG_BASE_EXCISION_REPAIR	0.3
KEGG_ONE_CARBON_POOL_BY_FOLATE	0.3
KEGG_RNA_DEGRADATION	0.34
KEGG_HOMOLOGOUS_RECOMBINATION	0.37
KEGG_CELL_CYCLE	0.49
KEGG_OOCYTE_MEIOSIS	0.55
KEGG_SPLICEOSOME	0.69
KEGG_PYRIMIDINE_METABOLISM	0.89
KEGG_NON_SMALL_CELL_LUNG_CANCER	0.89
KEGG_BLADDER_CANCER	1.23
KEGG_PURINE_METABOLISM	1.88
KEGG_CYTOSOLIC_DNA_SENSING_PATHWAY	1.92
KEGG_T_CELL_RECEPTOR_SIGNALING_PATHWAY	2.68
KEGG_ERBB_SIGNALING_PATHWAY	3.41
KEGG_NON_HOMOLOGOUS_END_JOINING	3.59
KEGG_CHRONIC_MYELOID_LEUKEMIA	3.79
KEGG_NOD_LIKE_RECEPTOR_SIGNALING_PATHWAY	3.93
KEGG_LYSINE_DEGRADATION	4.03
KEGG_B_CELL_RECEPTOR_SIGNALING_PATHWAY	4.24
KEGG_VEGF_SIGNALING_PATHWAY	4.45
KEGG_GLYCOSPHINGOLIPID_BIOSYNTHESIS_LACTO_AND_NEOLACTO_SERIES	4.52
KEGG_MISMATCH_REPAIR	4.61
KEGG_GLIOMA	4.65
KEGG_DORSO_VENTRAL_AXIS_FORMATION	4.67

KEGG_NATURAL_KILLER_CELL_MEDIATED_CYTOTOXICITY	4.82
**Gene Ontology Biological Process**	
GO_DNA_REPLICATION	<0.008
GO_POSITIVE_REGULATION_OF_CELL_CYCLE_PROCESS	<0.008
GO_MEIOTIC_CELL_CYCLE_PROCESS	<0.008
GO_DNA_CONFORMATION_CHANGE	<0.008
GO_POSITIVE_REGULATION_OF_CELL_CYCLE_PHASE_TRANSITION	<0.008
GO_DNA_DEPENDENT_DNA_REPLICATION	<0.008
GO_REGULATION_OF_CELL_CYCLE_PHASE_TRANSITION	<0.008
GO_POSITIVE_REGULATION_OF_CELL_CYCLE	<0.008
GO_DNA_RECOMBINATION	<0.008
GO_REGULATION_OF_CELL_CYCLE_G2_M_PHASE_TRANSITION	<0.008
GO_MRNA_EXPORT_FROM_NUCLEUS	<0.008
GO_CHROMOSOME_SEGREGATION	<0.008
GO_DNA_PACKAGING	<0.008
GO_ORGANELLE_FISSION	<0.008
GO_CELL_CYCLE_G2_M_PHASE_TRANSITION	<0.008
GO_NUCLEAR_CHROMOSOME_SEGREGATION	<0.008
GO_MITOTIC_NUCLEAR_DIVISION	<0.008
GO_POSITIVE_REGULATION_OF_MITOTIC_CELL_CYCLE	<0.008
GO_CELL_CYCLE_CHECKPOINT	<0.008
GO_SISTER_CHROMATID_SEGREGATION	<0.008
GO_MEIOSIS_I_CELL_CYCLE_PROCESS	<0.008
GO_SPINDLE_ORGANIZATION	<0.008
GO_MEIOTIC_CELL_CYCLE	<0.008
GO_REGULATION_OF_CYTOKINESIS	<0.008
GO_NEGATIVE_REGULATION_OF_CELL_CYCLE_PROCESS	<0.008
GO_MRNA_TRANSPORT	<0.008
GO_REGULATION_OF_CHROMOSOME_SEGREGATION	<0.008
GO_MITOTIC_CELL_CYCLE_CHECKPOINT	<0.008
GO_NEGATIVE_REGULATION_OF_CHROMOSOME_ORGANIZATION	<0.008
GO_CHROMOSOME_SEPARATION	<0.008
GO_MITOTIC_SPINDLE_ORGANIZATION	<0.008
GO_DOUBLE_STRAND_BREAK_REPAIR	<0.008
GO_MITOTIC_SISTER_CHROMATID_SEGREGATION	<0.008
GO_CELL_CYCLE_G1_S_PHASE_TRANSITION	<0.008
GO_TELOMERE_ORGANIZATION	<0.008
GO_MEIOTIC_CHROMOSOME_SEGREGATION	<0.008
GO_NUCLEAR_EXPORT	<0.008
GO_RECOMBINATIONAL_REPAIR	<0.008
GO_DNA_INTEGRITY_CHECKPOINT	<0.008
GO_DNA_GEOMETRIC_CHANGE	<0.008
GO_MICROTUBULE_CYTOSKELETON_ORGANIZATION_INVOLVED_IN_MITOSIS	<0.008
GO_DNA_REPAIR	<0.008
GO_NEGATIVE_REGULATION_OF_MITOTIC_CELL_CYCLE	<0.008
GO_CHROMOSOME_ORGANIZATION_INVOLVED_IN_MEIOTIC_CELL_CYCLE	<0.008
GO_REGULATION_OF_CHROMOSOME_ORGANIZATION	<0.008

GO_REGULATION_OF_CELL_CYCLE_ARREST	<0.008
GO_RNA_3_END_PROCESSING	<0.008
GO_RNA_EXPORT_FROM_NUCLEUS	<0.008
GO_PROTEIN_LOCALIZATION_TO_CYTOSKELETON	<0.008
GO_SIGNAL_TRANSDUCTION_INVOLVED_IN_CELL_CYCLE_CHECKPOINT	<0.008
