# Supplementary material for: Elevated expression of Aurora-A/AURKA in breast cancer associates with younger age and aggressive features
Source: Breast Cancer Res. 2024 Aug 28;26:126. doi: 10.1186/s13058-024-01882-x (PMC11360479; doi:10.1186/s13058-024-01882-x)
Supplement: Supplementary file 15 — Additional file 15. [file 13058_2024_1882_MOESM15_ESM.pdf]

Supplementary Table 9: Differentially expressed genes (DEGs) in ki67 high expression cases from the METABRIC combined cohorts: discovery and validation datasets with fold change ≥1.5/≤1.5 and false discovery rate (FDR) <0.008

	Ki67 high | METABRIC cohorts (n=1784)				
Upregulated DEGs			Downregulated DEGs		
Gene symbol	Fold change	FDR	Gene symbol	Fold change	FDR
CDC20	-3.11	<0.008	PIP	4.5	<0.008
UBE2C	-2.894	<0.008	AGR3	4.065	<0.008
SLC7A5	-2.442	<0.008	ANKRD30A	3.95	<0.008
CDCA5	-2.346	<0.008	TFF3	3.693	<0.008
BIRC5	-2.332	<0.008	NAT1	3.171	<0.008
CDCA7	-2.317	<0.008	SCGB2A2	3.063	<0.008
AURKB	-2.257	<0.008	ESR1	3.057	<0.008
FOXC1	-2.234	<0.008	FOXA1	3.021	<0.008
GABRP	-2.234	<0.008	C1ORF64	2.787	<0.008
ASPM	-2.219	<0.008	TFF1	2.783	<0.008
CBX2	-2.215	<0.008	SCGB1D2	2.718	<0.008
TOP2A	-2.184	<0.008	TBC1D9	2.674	<0.008
CCNB2	-2.183	<0.008	CA12	2.637	<0.008
PHGDH	-2.18	<0.008	SCUBE2	2.627	<0.008
C4ORF7	-2.154	<0.008	CLIC6	2.617	<0.008
KIF2C	-2.126	<0.008	MLPH	2.604	<0.008
CDC45L	-2.124	<0.008	MAPT	2.515	<0.008
FOXM1	-2.12	<0.008	CYP4X1	2.437	<0.008
E2F2	-2.116	<0.008	LOC646360	2.413	<0.008
BCL11A	-2.113	<0.008	DNAJC12	2.382	<0.008
KIFC1	-2.099	<0.008	SLC40A1	2.285	<0.008
PTTG1	-2.099	<0.008	LOC124220	2.282	<0.008
PSAT1	-2.094	<0.008	SLC7A2	2.262	<0.008
C1ORF106	-2.075	<0.008	HMGCS2	2.234	<0.008
MELK	-2.074	<0.008	REEP6	2.213	<0.008
PRC1	-2.064	<0.008	AZGP1	2.186	<0.008
TRIP13	-2.06	<0.008	SCNN1A	2.173	<0.008
MCM10	-2.059	<0.008	SUSD3	2.166	<0.008
CENPF	-2.059	<0.008	STC2	2.156	<0.008
KIF20A	-2.042	<0.008	NOSTRIN	2.148	<0.008
TPX2	-2.022	<0.008	GJA1	2.137	<0.008
TTK	-2.015	<0.008	SFRP2	2.132	<0.008
AURKA	-2.015	<0.008	THBS4	2.132	<0.008
TROAP	-2.013	<0.008	EEF1A2	2.12	<0.008
HJURP	-2.011	<0.008	POSTN	2.119	<0.008
LOC728715	-2.003	<0.008	SPARCL1	2.116	<0.008
NCAPG	-1.991	<0.008	GATA3	2.112	<0.008
CCNE1	-1.98	<0.008	SLC39A6	2.111	<0.008
EXO1	-1.975	<0.008	SFRP4	2.09	<0.008
CEP55	-1.962	<0.008	LOC644151	2.086	<0.008
CDCA8	-1.942	<0.008	C4ORF18	2.085	<0.008
CDCA3	-1.922	<0.008	PPP1R3C	2.084	<0.008

MCM2	-1.921	<0.008	CPA3	2.082	<0.008
KRT81	-1.918	<0.008	SEPP1	2.076	<0.008
LOC731049	-1.917	<0.008	APOD	2.07	<0.008
NUSAP1	-1.885	<0.008	CYBRD1	2.069	<0.008
CRABP1	-1.872	<0.008	AFF3	2.069	<0.008
CDC25B	-1.863	<0.008	NTN4	2.067	<0.008
CDT1	-1.859	<0.008	MS4A7	2.06	<0.008
MSLN	-1.857	<0.008	C9ORF152	2.06	<0.008
EIF2C2	-1.838	<0.008	CYP4Z1	2.056	<0.008
S100A8	-1.834	<0.008	LUM	2.053	<0.008
CENPA	-1.817	<0.008	SH3BGRL	2.051	<0.008
BUB1	-1.816	<0.008	DACH1	2.037	<0.008
PTTG3P	-1.811	<0.008	HS.388347	2.023	<0.008
C9ORF140	-1.8	<0.008	COL10A1	2.013	<0.008
CKAP2L	-1.799	<0.008	LRRC17	1.986	<0.008
TMSB15A	-1.796	<0.008	RNASE4	1.984	<0.008
POLQ	-1.794	<0.008	HS.570988	1.981	<0.008
CALML5	-1.794	<0.008	AGR2	1.978	<0.008
LOC651816	-1.784	<0.008	ELOVL5	1.974	<0.008
LAD1	-1.777	<0.008	CX3CR1	1.966	<0.008
KIF1A	-1.77	<0.008	FOS	1.964	<0.008
FAM64A	-1.763	<0.008	XBP1	1.956	<0.008
FEN1	-1.763	<0.008	CEACAM6	1.956	<0.008
TTYH1	-1.753	<0.008	CILP	1.927	<0.008
UHRF1	-1.749	<0.008	SERPINA3	1.905	<0.008
RARRES1	-1.745	<0.008	LOC389816	1.901	<0.008
STIL	-1.744	<0.008	PLAT	1.886	<0.008
DHCR7	-1.737	<0.008	HBB	1.883	<0.008
SPC24	-1.729	<0.008	DCN	1.882	<0.008
MCM4	-1.727	<0.008	ACOX2	1.881	<0.008
STMN1	-1.722	<0.008	RERG	1.879	<0.008
MX1	-1.721	<0.008	CYP4Z2P	1.871	<0.008
ASF1B	-1.715	<0.008	LRRC26	1.87	<0.008
C17ORF53	-1.707	<0.008	TCEAL1	1.863	<0.008
MCM7	-1.707	<0.008	MUCL1	1.842	<0.008
ELF5	-1.697	<0.008	CYB5A	1.84	<0.008
PROM1	-1.693	<0.008	C10ORF116	1.826	<0.008
CBS	-1.692	<0.008	TSPAN13	1.825	<0.008
SKP2	-1.69	<0.008	MYH11	1.823	<0.008
DSC2	-1.689	<0.008	CPB1	1.812	<0.008
CCNA2	-1.685	<0.008	HIST1H2AC	1.811	<0.008
TK1	-1.677	<0.008	CLEC3A	1.807	<0.008
HIST1H4C	-1.669	<0.008	TCN1	1.804	<0.008
ROPN1	-1.668	<0.008	HOXB2	1.798	<0.008
CENPE	-1.662	<0.008	GSTM2	1.798	<0.008
TYMS	-1.66	<0.008	SCGB2A1	1.792	<0.008
SOX11	-1.659	<0.008	DUSP1	1.791	<0.008
TACC3	-1.658	<0.008	CPE	1.782	<0.008

VGLL1	-1.657	<0.008	SPDEF	1.773	<0.008
CDH3	-1.652	<0.008	CMBL	1.771	<0.008
NCAPD2	-1.651	<0.008	TPSAB1	1.769	<0.008
LOC400578	-1.646	<0.008	CXCL12	1.762	<0.008
KNTC1	-1.643	<0.008	COX7A1	1.758	<0.008
LAMP3	-1.639	<0.008	OGN	1.752	<0.008
CXCL9	-1.639	<0.008	LOC389033	1.752	<0.008
RCOR2	-1.637	<0.008	SERPINA11	1.749	<0.008
SLC5A6	-1.637	<0.008	FCER1A	1.743	<0.008
TAP1	-1.637	<0.008	ECM2	1.742	<0.008
PFKP	-1.635	<0.008	PODN	1.74	<0.008
CENPN	-1.634	<0.008	FABP4	1.737	<0.008
KRT6B	-1.633	<0.008	PLAC9	1.735	<0.008
MGC102966	-1.632	<0.008	ADH1A	1.733	<0.008
DDX39	-1.63	<0.008	KIAA1370	1.731	<0.008
C6ORF173	-1.629	<0.008	GFRA1	1.727	<0.008
RAD51AP1	-1.627	<0.008	FSIP1	1.726	<0.008
HMGA1	-1.622	<0.008	SMOC2	1.724	<0.008
PKP1	-1.621	<0.008	GP2	1.724	<0.008
RECQL4	-1.619	<0.008	CTSG	1.717	<0.008
FAM83D	-1.618	<0.008	C10ORF32	1.713	<0.008
LMNB2	-1.615	<0.008	HTRA1	1.704	<0.008
CENPM	-1.615	<0.008	PDZK1	1.699	<0.008
ART3	-1.615	<0.008	HS.389988	1.698	<0.008
GTSE1	-1.611	<0.008	C14ORF45	1.697	<0.008
EZH2	-1.611	<0.008	SPARC	1.697	<0.008
LAG3	-1.609	<0.008	CCL15	1.697	<0.008
KIF4A	-1.601	<0.008	MFAP4	1.697	<0.008
CXCL10	-1.6	<0.008	DNALI1	1.697	<0.008
KIF14	-1.598	<0.008	KIAA1324	1.694	<0.008
KIF15	-1.598	<0.008	FAM134B	1.692	<0.008
LRP8	-1.598	<0.008	MUC1	1.692	<0.008
BOP1	-1.598	<0.008	CCNDBP1	1.689	<0.008
SKA1	-1.596	<0.008	CFB	1.686	<0.008
MCM3	-1.594	<0.008	LRG1	1.685	<0.008
C1ORF135	-1.594	<0.008	OMD	1.681	<0.008
NEK2	-1.592	<0.008	SYTL2	1.676	<0.008
BLM	-1.59	<0.008	DKK3	1.675	<0.008
GPT2	-1.59	<0.008	F13A1	1.675	<0.008
RACGAP1	-1.589	<0.008	REEP1	1.675	<0.008
NDRG1	-1.589	<0.008	CRIP1	1.674	<0.008
UBE2T	-1.587	<0.008	QDPR	1.673	<0.008
TTLL4	-1.586	<0.008	LTF	1.673	<0.008
MCM6	-1.583	<0.008	PDGFRL	1.672	<0.008
MCM5	-1.581	<0.008	GOLSYN	1.672	<0.008
C18ORF56	-1.581	<0.008	C20ORF103	1.671	<0.008
ROPN1B	-1.578	<0.008	GSTM1	1.665	<0.008

NFIB	-1.578	<0.008	FGD3	1.658	<0.008
KIF11	-1.577	<0.008	HS.7413	1.656	<0.008
KRT16	-1.577	<0.008	RTN1	1.652	<0.008
PKMYT1	-1.576	<0.008	ALCAM	1.651	<0.008
NMU	-1.575	<0.008	GPR160	1.647	<0.008
STAT1	-1.573	<0.008	MGP	1.646	<0.008
NT5DC2	-1.572	<0.008	MYB	1.639	<0.008
CDCA2	-1.571	<0.008	AGTR1	1.636	<0.008
LOC731314	-1.57	<0.008	HS.573062	1.634	<0.008
LCN2	-1.57	<0.008	LOC338579	1.631	<0.008
CTSL2	-1.569	<0.008	CFD	1.63	<0.008
NOP2	-1.568	<0.008	PKIB	1.63	<0.008
MKI67	-1.565	<0.008	FOSB	1.627	<0.008
FANCD2	-1.563	<0.008	HIGD1A	1.626	<0.008
MMP9	-1.559	<0.008	RGS5	1.626	<0.008
GZMB	-1.558	<0.008	ENPP5	1.626	<0.008
SOX10	-1.557	<0.008	SERPINA1	1.626	<0.008
FZD9	-1.549	<0.008	GSTT1	1.626	<0.008
GPSM2	-1.548	<0.008	HS.25318	1.623	<0.008
OIP5	-1.544	<0.008	GNG11	1.622	<0.008
HS.579631	-1.544	<0.008	CAPN13	1.622	<0.008
KIF20B	-1.543	<0.008	C9ORF116	1.621	<0.008
CDC25A	-1.539	<0.008	PRDX3	1.615	<0.008
RASD2	-1.537	<0.008	ARMCX1	1.614	<0.008
PBK	-1.536	<0.008	CDH11	1.614	<0.008
A2ML1	-1.535	<0.008	MFAP5	1.614	<0.008
CLCC1	-1.529	<0.008	CTSK	1.613	<0.008
PPP1R14C	-1.528	<0.008	BMP4	1.612	<0.008
S100A9	-1.526	<0.008	GPR177	1.611	<0.008
KIF23	-1.524	<0.008	ITPR1	1.607	<0.008
DLGAP5	-1.522	<0.008	C4ORF32	1.603	<0.008
IGF2BP2	-1.522	<0.008	PSD3	1.6	<0.008
HERC5	-1.52	<0.008	UGT2B11	1.599	<0.008
KRT86	-1.519	<0.008	FMO5	1.598	<0.008
C15ORF42	-1.518	<0.008	TGFB3	1.594	<0.008
E2F3	-1.518	<0.008	HS.144479	1.594	<0.008
CDKN3	-1.516	<0.008	C4B	1.592	<0.008
PSRC1	-1.515	<0.008	RHOB	1.592	<0.008
SMC4	-1.515	<0.008	FMOD	1.591	<0.008
CEBPB	-1.513	<0.008	UGDH	1.587	<0.008
ARL9	-1.51	<0.008	ZFAND6	1.584	<0.008
GBP5	-1.51	<0.008	SUB1	1.583	<0.008
ATAD2	-1.509	<0.008	DARC	1.583	<0.008

FAM171A1	-1.509	<0.008	LOC388743	1.583	<0.008
GAL	-1.509	<0.008	CIRBP	1.579	<0.008
WARS	-1.508	<0.008	TPM1	1.579	<0.008
UCK2	-1.506	<0.008	LRIG1	1.578	<0.008
HAPLN3	-1.505	<0.008	TAT	1.578	<0.008
RNASEH2A	-1.504	<0.008	GNG12	1.574	<0.008
			LOC375295	1.574	<0.008
			FAM174A	1.573	<0.008
			EFEMP1	1.573	<0.008
			LOC388588	1.572	<0.008
			NME5	1.571	<0.008
			GLRB	1.571	<0.008
			TIMP3	1.57	<0.008
			C6ORF211	1.57	<0.008
			TSPAN1	1.57	<0.008
			LOC389787	1.569	<0.008
			COL3A1	1.569	<0.008
			HS.159264	1.569	<0.008
			ACOT4	1.568	<0.008
			DIO1	1.568	<0.008
			SPON1	1.567	<0.008
			LOC145837	1.567	<0.008
			HBA2	1.567	<0.008
			RARRES3	1.565	<0.008
			TNFSF10	1.564	<0.008
			RPL21	1.562	<0.008
			SEC14L2	1.561	<0.008
			STK32B	1.561	<0.008
			C8ORF4	1.561	<0.008
			COL1A2	1.56	<0.008
			LOC647673	1.559	<0.008
			C4ORF34	1.558	<0.008
			PTPRT	1.558	<0.008
			IGFBP4	1.556	<0.008
			GLT8D2	1.554	<0.008
			AKR7A3	1.554	<0.008
			IGJ	1.554	<0.008
			PECI	1.553	<0.008
			LOC651149	1.553	<0.008

DYNLRB2	1.551	<0.008
MATN3	1.551	<0.008
LOC402251	1.551	<0.008
PGR	1.55	<0.008
VAV3	1.55	<0.008
GRPR	1.548	<0.008
RABEP1	1.547	<0.008
BTG2	1.546	<0.008
EGR1	1.543	<0.008
CTSO	1.541	<0.008
PTGR1	1.541	<0.008
KLK4	1.538	<0.008
MGC87042	1.538	<0.008
DEGS2	1.538	<0.008
ANXA9	1.537	<0.008
STEAP2	1.536	<0.008
CXCL14	1.536	<0.008
CAV1	1.533	<0.008
SNURF	1.533	<0.008
FBLN1	1.532	<0.008
RPS23	1.532	<0.008
UGT2B7	1.532	<0.008
SLC12A2	1.53	<0.008
NUCB2	1.529	<0.008
GAMT	1.528	<0.008
MEIS3P1	1.527	<0.008
KIF13B	1.527	<0.008
SC5DL	1.524	<0.008
ANG	1.524	<0.008
SVEP1	1.524	<0.008
LOC644063	1.523	<0.008
SOCS2	1.523	<0.008
GNG10	1.522	<0.008
BEX4	1.521	<0.008
THRA	1.521	<0.008
CRTAP	1.519	<0.008
COMMD10	1.512	<0.008
FBP1	1.512	<0.008
FHL2	1.51	<0.008
GALNT10	1.507	<0.008
GPR81	1.507	<0.008
FAP	1.506	<0.008
NDN	1.505	<0.008
PMP22	1.504	<0.008

CIDEC	1.504	<0.008
CLDN11	1.503	<0.008
ACOT1	1.502	<0.008
CAMK2N1	1.501	<0.008
BEX1	1.5	<0.008
